# Silencing BRE Expression in Human Umbilical Cord Perivascular (HUCPV) Progenitor Cells Accelerates Osteogenic and Chondrogenic Differentiation

**DOI:** 10.1371/journal.pone.0067896

**Published:** 2013-07-23

**Authors:** Elve Chen, Mei Kuen Tang, Yao Yao, Winifred Wing Yiu Yau, Lok Man Lo, Xuesong Yang, Yiu Loon Chui, John Chan, Kenneth Ka Ho Lee

**Affiliations:** 1 Stem Cell and Regeneration Thematic Research Programme, School of Biomedical Sciences, Chinese University of Hong Kong, Hong Kong, People's Republic of China; 2 Key Laboratory for Regenerative Medicine Ministry of Education, Jinan University, Guangzhou, People's Republic of China; 3 Department of Chemical Pathology, Chinese University of Hong Kong, Chinese University of Hong Kong, Hong Kong, People's Republic of China; 4 School of Pharmacy and Life Sciences, Robert Gordon University, Aberdeen, Scotland, United Kingdom; Centro Cardiologico Monzino, Italy

## Abstract

BRE is a multifunctional adapter protein involved in DNA repair, cell survival and stress response. To date, most studies of this protein have been focused in the tumor model. The role of BRE in stem cell biology has never been investigated. Therefore, we have used HUCPV progenitor cells to elucidate the function of BRE. HUCPV cells are multipotent fetal progenitor cells which possess the ability to differentiate into a multitude of mesenchymal cell lineages when chemically induced and can be more easily amplified in culture. In this study, we have established that BRE expression was normally expressed in HUCPV cells but become down-regulated when the cells were induced to differentiate. In addition, silencing *BRE* expression, using *BRE*-siRNAs, in HUCPV cells could accelerate induced chondrogenic and osteogenic differentiation. Hence, we postulated that BRE played an important role in maintaining the stemness of HUCPV cells. We used microarray analysis to examine the transcriptome of *BRE*-silenced cells. *BRE*-silencing negatively regulated *OCT4*, *FGF5* and *FOXO1A*. *BRE*-silencing also altered the expression of epigenetic genes and components of the TGF-β/BMP and FGF signaling pathways which are crucially involved in maintaining stem cell self-renewal. Comparative proteomic profiling also revealed that *BRE*-silencing resulted in decreased expressions of actin-binding proteins. In sum, we propose that BRE acts like an adaptor protein that promotes stemness and at the same time inhibits the differentiation of HUCPV cells.

## Introduction

BRE is also known as TNFRSF1A modulator and BRCC45. This highly conserved gene was initially identified as a stress-responsive gene – whose expression was inhibited following DNA damage and retinoic acid treatment [1]. BRE shares no homology with other known gene products [2]. The protein contains two putative ubiquitin E2 variant domains but lacks critical cysteine which is required for ubiquitination [3]. BRE protein is present in both the cytosol and nucleus. In the cytoplasm, BRE binds to the cytoplasmic region of p55 TNF receptors to suppress TNF-α induced activation of NF-κB [4]. The protein can also bind to Fas to inhibit the mitochondrial apoptotic pathway [5]. BRE is also found to be a component in BRISC complex that specifically cleaves lysine63-linked ubiquitin [6]. BRE may function as a key adaptor protein which assembles the different components of the BRISC complex [7]. In the nucleus, BRE is a component of the DNA damage responsive BRCA1-RAP80 complex. BRE protein acts as an adapter that links the interaction between NBA1 and the rest of the complex. This adapter modulates the ubiquitin E3 ligase activity of the BRCA1/BARD1 complex by interacting with MERIT 40 which enhances cellular survival following DNA damage [7], [8], [9].

BRE has been extensively studied in lung tumour, hepatocellular carcinoma and oesophageal carcinoma [10], [11], [12], [13] - showing that it promoted cell survival. Nevertheless, the function of BRE in stem cells has never been investigated. BRE is expressed very early on in embryonic development; at the 2-cell stage, in the inner cell mass cells of blastocysts and even in embryonic stem cells. In this context, we want to establish why BRE was expressed so early in development and whether it was involved in maintaining stemness and cell differentiation. It is generally accepted that stress-responsive genes, like BRE, play a crucial role in biological processes such as cell survival, differentiation, apoptosis and regeneration. To address our questions, we employed HUCPV progenitor cells as our experimental cell model [14]. These cells are normally found in the perivascular regions of human umbilical arteries and vein and contain a rich source of commercially valuable mesenchymal stem cells (MSCs). The HUCPV cells were found to have a colony forming unit-fibroblast (CFU-F) frequency of about 1∶300, which is far higher than that of bone marrow (1∶10^4^–1∶10^6^, depending on age) or umbilical cord blood (1∶200 million). The HUCPV cells also showed a higher proliferative potential and expressed higher levels of CD146 (a putative MSC marker) in comparison to MSCs obtained from bone marrow [15]. These cells also express surface antigens CD44, CD73, CD90, CD105 and CD106 but do not express CD34 or CD45 [14], [15], [16]. HUCPV cells are multipotent and capable of differentiating into all mesenchymal lineages *in vitro* [15], [16], [17]. Notably, these cells contribute to rapid connective tissue healing *in vivo* by producing bone and fibrous stroma [18]. Besides being multipotent, these cells are immunoprivileged making them less likely to be immune-rejected as allografts [16], [19]. Furthermore, these foetal cells express the embryonic cell markers SSEA-4, RUNX1 and OCT4 [20]. HUCPV cells can be obtained non-invasively, making them an ideal source for stem cells therapies.

## Materials and Methods

### Cell culture

**Figure 1:**
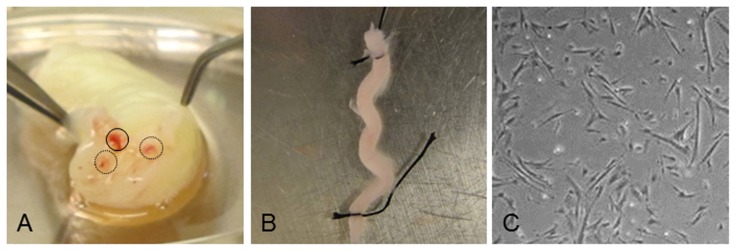
Extraction and purification of HUCPV cells. (A) Representative picture of a human umbilical cord showing the umbilical vein (represented by solid circle) and umbilical arteries (represented by dashed circle). (B) Prior to treatment with collagenase, the umbilical blood vessel was ligated at both ends. (C) The primary HUCPV cells were isolated by collagenase digestion of the perivascular region of the ligated blood vessel.

The human umbilical cords were obtained from the Department of Obstetrics and Gynecology and were approved by Chinese University of Hong Kong Clinical Research Ethics committee (project reference number CRE 2011.116). This is centrally registered with the Hong Kong Health Authority. The physician obtained verbal informed consent from the mother for use of the umbilical cord in research. The cords were then dissected aseptically with the aid of a dissecting microscope as described by Sarugaser et al. [16]. Briefly, the amniotic epithelium was removed from the cord with forceps and scissors (Figure 1A). The two umbilical arteries and umbilical vein were then separated from the cord using forceps. The vessels were then tied in a loop at each ends using sutures as shown in Figure 1B and the placed in 80 U/mL type I collagenase (Gibco) and 0.01 U/mL in a 50 mL Falcon tube. The digestion was carried out in a shaker for four hours at 37°C. The extracted cells were then centrifuged at 500 rpm for 5 min at room temperature. The supernatant was next centrifuged at 1,500 rpm for 3 min at room temperature. The pellet of cells were resuspended in regular growth medium consists of DMEM/F12 supplemented with 15% embryonic stem cell-qualified fetal bovine serum (ESQ-FBS), 100 units/mL penicillin and 100 µg/mL streptomycin (all from Gibco) and seeded into culture dish coated with 1% gelatin in ddH_2_O. The cells are maintained in a 5% CO_2_ humidified incubator (Thermo Scientific). After one week culture, the isolated HUCPV progenitor cells became confluent for the analysis (Figure 1C).

### Flow cytometry

The crude HUCPV progenitor cells were purified by flow cytometry. Briefly, the confluent culture was trypsinized into suspension and incubated with anti-human CD44, CD90, CD105 and CD146 conjugated PE antibodies for positive selections and anti-human CD34 and CD45 conjugated PE antibodies for negative selection. All antibodies were purchased from BD Biosciences. The immune reactions were performed at 4°C for 20 min. The cells were analyzed and sorted using a FACSAria flow cytometer (BD Biosciences) with FACSDiva software (BD Biosciences).

### Mouse embryonic stem cell (ESC) culture

Mouse ES cell line (AINV15, obtained from ATTC) was cultured on 13 mm glass coverslips in 1,400 U/ml of LIF (Millipore) and expanded by co-culture with 10 µg/ml mitomycin C-inactivated mouse embryonic fibroblasts to inhibit differentiation. To induce ESC differentiation, LIF was withdrawn from the culture medium for 24 hours and then the cells were fixed in 10% formalin. Along with undifferentiated ESC cultures, they were processed for immunofluorescent staining with BRE and OCT4 antibodies. Each immunofluorescent staining analysis was performed in triplicate.

### Immunofluorescence microscopy

HUCPV cells, cultured on glass coverslips, were fixed in 10% formalin and permeabilized with 0.5% Triton X-100 (Sigma) with 0.1% SDS (Sigma) for 30 min. The samples were then washed three times with PBS and blocked with 2% BSA with 5% normal horse serum for 1 hour. Afterward, the samples were incubated with primary antibody overnight. Primary antibodies used in this study include: CD146 (Zymed, Invitrogen), Ki-67 (Santa Cruz), SOX9 (Abcam), type I collagen (Millipore), type II collagen (Millipore). Non-specific antibody binding were then washed with PBS with 0.05% Tween-20 (PBST) three times for 10 min and PBS for 5 min. Then secondary antibody (Jackson ImmunoResearch Laboratories) was added and incubated for 1 hour. The unbound antibodies were washed with PBST three times for 10 min and PBS for 5 min. DAPI was added for visualizing the nucleus. The fluorescent signal was detected using a confocal microscope (Olympus, FluoView 1000) under a Fluequipped with 40× Zeiss PlanNeofluo objectives.

### RT-qPCR and statistical analysis

Total RNA was isolated from cells using TRIzol® reagent (Invitrogen) according to the manufacturer's instruction. The purity, integrity, and concentrations of RNA were evaluated using the Nanodrop Bioanalyzer (Nanodrop Technologies). First strand cDNA was obtained from 1 µg total RNA reverse transcribed using Ready-To-Go You-Prime First-Strand beads and oligo(dT) following the manufacturer's instruction (GE Healthcare). The resulting cDNAs were amplified using SYBR® Premix Ex Taq mix (Takara Biotechnology) and analyzed by a 7900HT Fast Real-Time PCR System (Applied Biosystems). The shuttle PCR protocol included initial denaturation (95°C for 30 second), annealing (40 cycles of 95°C for 5 second and 60°C for 30 second) and final extension (95°C for 15 second, 60°C for 1 minute, and 95°C for 15 second). Genes of interest were analyzed and normalized against house-keeping gene, GAPDH. The other internal house-keeping gene, glucuronidase-β (gusb) was selected to check the stability of GAPDH. Experiments were performed in triplicates. The expression levels were calculated by 2^−ΔΔCT^ method [21]. For Statistical analysis, the data was presented as mean ± standard deviation. Statistical significance was determined by Student's t-test. The sequences of the primer sets used for the RT-qPCR reactions are listed in Table S1. The primers were designed with PrimerDepot database (http://primerdepot.nci.nih.gov/) or Primer3Plus software (http://www.pubmed.de/cgi-bin/primer3/primer3plus.cgi).

### Transfection with siRNA in HUCPV cells

Transient BRE silencing was performed using siRNAs and Lipofectamine™ RNAiMAX (Invitrogen) transfection reagent. The transfection was conducted following the manufacturer's protocol using 12 pmol of siRNA together with 1 µL transfection reagent in Opti-MEM no serum medium (Invitrogen). The target sequence of *BRE*-siRNA was: AAC TGG ACT GGT GAA TTT TCA. The control (Ctl) is a scrambled siRNA was purchased from Healthcare and Co., Hong Kong. The experiments were performed in triplicate.

### Microarray analysis

Total RNA was extracted from HUCPV cells using NucleoSpin® RNA II (Macherey-Nagel) according to manufacturer's instructions. The RNA integrity was determined by Nimblegen bioanalyzer prior to microarray hybridization (Roche). Then, 10 µg of total RNA was reverse transcribed by SuperScript™ Double-Stranded cDNA Synthesis Kit (Invitrogen). For Cy3 cDNA labelling, 1 µg of double-stranded cDNA sample was labelled using the NimbleGen One-Color DNA labelling kit (Roche) following the manufacturer's manual. Briefly, 1 µg cDNA was labelled with Cy3 random nanomers using Klenow fragment. Followed by incubation at 37°C for 2 hours, the labelling reaction was next terminated using EDTA. After isopropanol precipitation, the Cy3 labelled cDNA pellet was rinsed with 80% ethanol and vacuum dried on low heat. Cy3 labelled cDNA was rehydrated in 25 µl PCR grade water and quantified using Quan-iT PicoGreen dsDNA assay kit (Invitrogen). The analysis was performed in triplicate.

Array hybridization was performed on NimbleGen human 12× 135K v5.1 high-density single channel oligonucleotide arrays (Roche). Briefly, 4 µg of Cy3 labelled cDNA was vacuumed dried and resuspended in 3.3 µl sample tracking controls and hybridized at 42°C in NumbleGen Hybrization System 4 for 16 hours. Next, the array was washed using the NimbleGen wash buffer kits, the array slide was spin dried and scanned using the NimbelGen MS 200 Microarray Scanner. Scanned images were analyzed using a DEVA software Version 1.0.1 (Roche) and Partek Genomics Suite version 6.12.0103 (Partek). Array data has been deposited in NCBI Gene Expression Omnibus (GEO, http://www.ncbi.nlm.nih.gov/geo/) number GSE39948.

### Comparative Proteomics

Total protein lysates were extracted from HUCPV cells transfected with *BRE*-siRNA or *Ctl-siRNAs*. Extraction condition, two-dimensional gel electrophoresis (2-DE), MALDI-TOF mass spectrometry and bioinformatics search were performed as we previously reported [22], [23], [24]. Briefly, first dimensional electrophosis (1-DE) was performed on an IPGphor IEF system using 11-cm long IPG electrode strip with pH 3–10 gradient (Amersham Biosciences, UK) and an Ettan IPGphor Strip Holder (Amersham Biosciences, UK). 150 µg of protein was applied for each IPG strip. The total volume of protein sample with rehydration buffer (8M Urea, 2% CHAPS (w/v), 1% IPG buffer (v/v), 40 mM DTT) loaded onto the strip holder was 210 µl. The sufficient volume of IPG Cover Fluid was applied to each strip so as to minimize evaporation and urea crystallization. The rehydration step was done under voltage and followed by a separation process. The electrophoresis condition for step 1 was 30 V for 13 hrs; step 2 was 500 V for 1 hr; step 3 was 2000 V for 1 hr and step 4 was 5000 V for 20 hrs. The program was stopped when the total volt-hours reached 40000. After the 1-DE was completed, the sample strips were removed from the strip holders. Each strip was then treated with 1% DTT in 6.5 ml of equilibration buffer (50 mM Tris, 6M of urea, 30% glycerol, 2% SDS, 0.1% bromophenol blue) for 30 min. The medium was then changed to 1% iodoacetamide (IAA, w/v, Sigma-Aldrich, USA) dissolved in the 6.5 ml of the same equilibration buffer. The strips were treated in the solution for 30 min and then loaded onto 12% SDS-polyacrylamide gels with 0.2% agarose in electrophoresis running buffer (25 mM Tris, 192 mM glycine, 0.1%SDS, adjust to pH 8.3). Protein markers (20 to 120 kDa, Fermentas Life Sciences) were also loaded into the gel for determining the size of all the proteins resolved in the gel. The 2-DE was performed in an ISO-DALT apparatus (Hoefer Scientific Instruments) at room temperature under constant voltage 100 V till the dye front reached the bottom of the gel. The gels were then fixed in 50% methanol, 12% acetic acid and 0.5 ml 37% formaldehyde for 1 hour. After fixation, the gels were washed in MilliQ water (4×), 50% ethanol (v/v) for 20 min (2×), 0.02% sodium thiosulphate (w/v, Merck, UK) for 10 min and distilled water (3×). Subsequently, the gel was stained in silver solution (0.15% silver nitrate in 0.75 ml 37% formaldehyde) at 4°C for 1 hr. After several brief washes, the gels were developed in developer solution (1 ml 37% formaldehyde, 30 g sodium carbonate and 2 mg sodium thiosulphate in one liter buffer) until the desired staining intensity was attained. The gels were then immersed in 5% acetic acid (v/v, BDH Chemicals Ltd., UK) for 5 mins to terminate the staining process. Finally, the silver stained gels were scanned using a GS 800 Densitometer (Bio-Rad Laboratories, USA) and the images captured were used for image analysis. The protein spots on the gel were analysed using a PDQuest 2D Analysis Software version 7.13 PC (The Discovery Series, Bio-Rad Laboratories, USA). Each experiment was performed in triplicate.

### Protein identification by mass fingerprinting

All protein spots of interest were isolated from the 2D-gel and processed for silver destaining. The gel pieces were first washed in MilliQ water, immersed in 200 µl of destaining solution (15 mM potassium ferricyanide and 50 mM sodium thiosulphate) and then incubated at room temperature until they turned colourless. Each gel pieces was then washed in 400 µl of MilliQ water for 15 min (3×). The destained gel pieces were then equilibrated in 200 µl of 10 mM ammonium bicarbonate/50% acetonitrile for 15 min. The gel was dehydrated in 200 µl of acetonitrile for 15 min and dried at 30°C for 5 min. The gels were digested with 15 µg/ml of trypsin in 40 mM ammonium bicarbonate/50% acetonitrile (v/v) at 35°C for 16 hrs. Three µl of extraction solution (50% acetonitrile (v/v) and 5% trifluoroacetic acid (Fluka Chemika, Switzerland) were used to stop the reaction. Three µl of reaction mixture was mixed with α-cyano-4-hydroxycinnamic acid matrix and then spotted onto a sample plate for ESI-MS/MS analysis (Bruker Daltonics, USA). The mass spectrums generated were analysed using a Bruker Daltonics software and by mass fingerprinting, which were submitted to the SwissPort bioinformation stations using MASCOT 2.2.07 engine search.

### Immunoprecipitation (IP) Assay

*Ctl-siRNAs* and *BRE-siRNA* treated cell samples were collected and lysed by in denature buffer (50 mM Tris pH 8.0, 150 mM NaCl, 1% NP-40 and 0.5% sodium deoxycholate) inside a microcentrifuge tube. After the samples were centrifuged for 20 mins at 12,000 rpm at 4°C, the supernatant were aspirated and place in a fresh tube. The lysates were pre-cleared with Sepharose beads for 1 hr on ice and spun in centrifuge to discard the bead pellet. The pre-clear lysates were incubated with the appropriate concentration of antibody in the Sepharose beads, overnight. When the incubation was completed, the lysates were washed in lysis buffer 3×. Finally, the lysates were added the 2× loading buffer and boiled at 95°C for 5 mins. The lysate samples were resolved and the proteins were analysed by routine western blot analysis using the Odyssey® CLx infrared imaging system. The actin, ANAXII and BRE were used for the IP assay. The Assay was performed in triplicate.

### Scratch cell migration assay

The scratch assay was performed according to methods described by Liang et al. [56]. The *Ctl-siRNAs* and *BRE-siRNA* treated HUCPV cells were seeded onto 4-well culture plates. The cells were then treated with 2 µg/ml mitomycin C (Sigma, USA) for 1 hr to prevent further cell proliferation (which could confound our interpretation of the cell migration analysis). After the treatment, a sterile p10 pipet tip was used to scrap off cells from the center of the monolayer to create a gap in the culture. Reference lines were etched onto the bottom of the plastic culture dishes to define the position of the gap/wound. Photos were taken of the *Ctl-siRNAs* and *BRE-siRNA* treated cells migrating into the wound area at day 0–day 3 incubation. The photographic images captured were quantitatively analysed by establishing the number of cells that have migrated into the gap. The assay was performed in triplicate.

## Results

### HUCPV cells are multipotent

**Figure 2:**
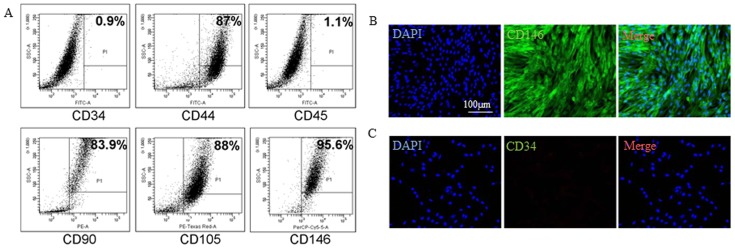
Purification of HUCPV cells. (A) HUCPV cells were analyzed with surface markers which included CD34, CD44, CD45, CD90, CD105 and CD146. (B and C) Immunofluorescence staining confirmed that the HUCPV cells expressed CD146 but not CD34. The nuclei were counterstained with DAPI.

HUCPV cells are normally found in the perivascular regions of human umbilical arteries and veins, which we isolated according to procedures described by Sarugaser et al. [16]. Briefly, both ends of the umbilical vessels were first ligated and then the perivascular region containing the HUCPV cells was digested using collagenase (Figures 1A–C). The crude cell extracts produced were then sorted and purified for HUCPV cells using flow cytometery for CD34^−^, CD45^−^, CD44^+^, CD90^+^, CD105^+^ and CD146^+^ surface markers (Figure 2A). Immunofluorescence staining revealed that our HUCPV cells strongly expressed CD146, which is a marker for mesenchymal stem cell (Figure 2B). The cells were negatively stained for CD34, which is a hematopoietic stem cell marker (Figure 2C).

**Figure 3:**
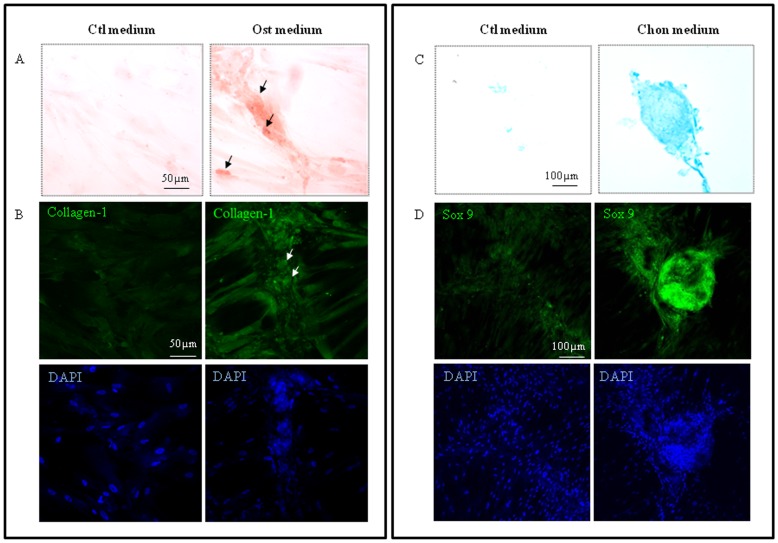
HUCPV cells differentiated into osteoblasts and chondrocytes, 3 and 4 weeks after induction, respectively. (A) Alizarin red S staining and immunofluorescence staining with pro-collagen type-I antibody were used to demonstrate osteogenic differentiation by HUCPV cells maintained in osteogenic (Ost) medium and control (Ctl) media for 3 weeks. (B) Alcian blue staining and immunofluorescence staining with SOX9 antibody were used to demonstrate chondrogenic differentiation in HUCPV cells cultured in chondrogenic (Chon) and control (Ctl) media for 4 weeks. The nuclei were counterstained with DAPI. N = 3 independent experiments.

To assess the developmental potential of HUCPV cells, they were treated with osteogenic and chondrogenic inducing media. These cells were able to differentiate into osteoblasts after 3 weeks induction. Alizarin red S staining revealed the presence of calcified bone matrix in the cell culture as the HUCPV cells underwent osteogenesis (Figure 3A). These osteogenic induced HUCPV cells also expressed collagen type I while the control cultures only expressed background levels of collagen (Figure 3B). Similarly, the HUCPV cells were tested for their chondrogenic potential and were treated with chondrogenic induction medium. Alcian blue staining revealed that the induced HUCPV cells deposited sulfated proteoglycans 4 weeks after treatment (Figure 3C). These cells also expressed the key transcription factor SOX9 and formed cell aggregates not found in control cultures (Figure 3D).

### BRE expression during HUCPV and embryonic stem cell (ESC) differentiation

**Figure 4:**
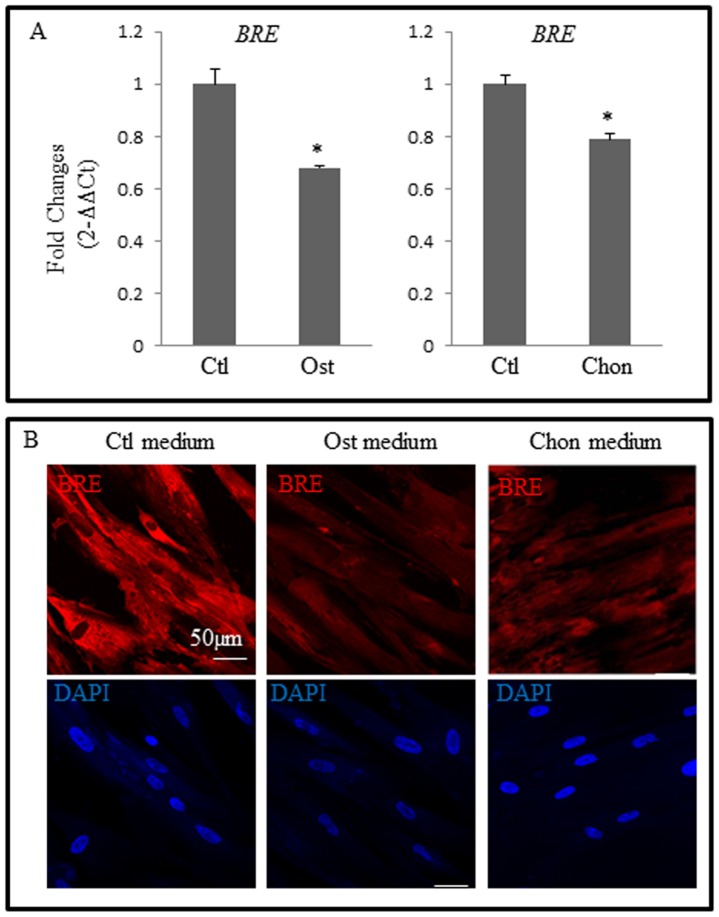
BRE expressions during HUCPV cell differentiation. (A) RT-qPCR revealed that as HUCPV cells were induced to differentiate in osteogenic (Ost) and chondrogenic (chon) medium, *BRE* expression was down-regulated. *BRE* expression was normalized against *GAPDH*. The statistical difference of P values were determined by t-test; *p<0.05, **p<0.01 and *p<.05 were considered significantly different. (B) Immunofluorescence microscopy confirmed that BRE expression (red) was suppressed as the HUCPV cells were induced to form osteoblasts and chondroblasts. Control (Ctl) medium. Osteogenic (Ost) and chondrogenic (Chon) inducing medium. The nuclei were counterstained with DAPI. N = 3 independent experiments.

It has been reported that *BRE* expression was down-regulated when tumor cells were treated with retinoic acid, a differentiating agent [1]. Therefore, we examined *BRE* expression in HUCPV cells when they are induced to differentiate. We established that *BRE* expression was down-regulated when HUCPV cells were induced in osteogenic medium after 3 weeks or chondrogenic medium after 4 weeks as compared with control medium (Figure 4A). Immunofluorescence staining using BRE antibody also confirmed that BRE protein expression was decreased as HUCPV cells differentiated into osteoblasts and chondrocytes (Figure 4B). Therefore, we hypothesize that BRE suppresses HUCPV cell differentiation.

**Figure 5:**
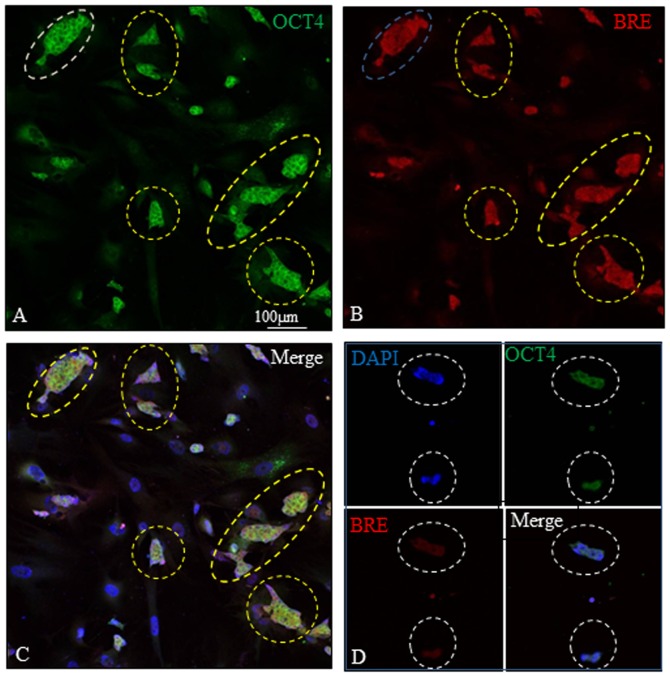
BRE and OCT4 expression in ESCs. (A–C) In the presence of LIF, BRE and OCT4 were strongly co-expressed in undifferentiated ESCs (yellow dotted outlines). (D) LIF was withdrawn from the culture for 24 hours to allow the ESCs to differentiate. This resulted in a reduction of BRE and OCT4 expression in the ESCs (white dotted outlines). N = 3 independent experiments.

Besides HUCPV cells, we also examined the co-localization of Oct4 (pluripotent marker) and BRE in mouse embryonic stem cells (ESCs). In the presence of LIF, we found that BRE and OCT4 were strongly co-expressed in undifferentiated ESCs (Figure 5 A–C). When LIF was withdrawn from the culture medium to allow the ESCs to differentiate, it resulted in a synchronized reduction of BRE and OCT4 expression (Figure 5D).

### Silencing BRE accelerates chondrogenic and osteogenic induction

**Figure 6:**
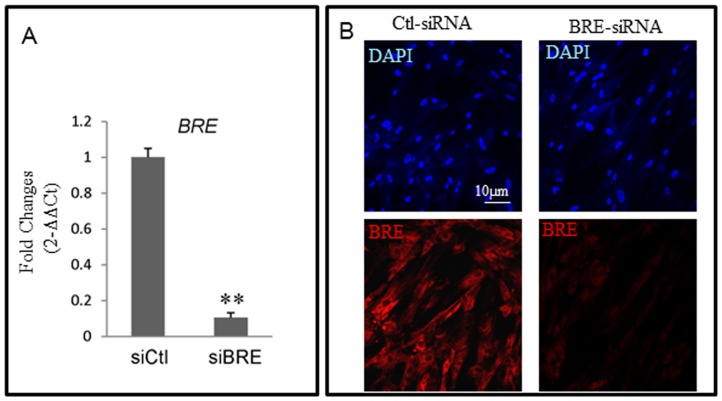
BRE expressions in *BRE*-silenced HUCPV cells. (A) RT-qPCR and (B) immunofluorescence microscopy showing *BRE*-siRNAs could silence BRE expression in HUCPV cells. Our control *Ctl-siRNAs* did not affect BRE expression. For RT-qPCR, *BRE* expression was normalized to housekeeping gene *GAPDH*. The statistical difference of P values were determined by t-test and **p<0.01 were considered significantly different. The nuclei were counterstained with DAPI. N = 3 independent experiments.

We have used siRNAs to silence *BRE* expression to test our hypothesis that BRE inhibits HUCPV cell differentiation. HUCPV cells were transfected twice with either *BRE*- or *ctl*- (scrambled control) siRNAs at 24-hour intervals, prior to inducing the cells with differentiation medium. We first confirmed that transfection with our *BRE*-siRNA was able to significantly reduce both BRE mRNA and protein expressions in HUCPV cells (Figure 6A). In addition, the growth rate and survival rate of *BRE*-silenced HUCPV cells were unaffected when compared with the control. This is evident by the duration required for *BRE*-silenced and control cells to reach confluency and the total number of DAPI labeled cells present in the cultures (Figure 6B).

**Figure 7:**
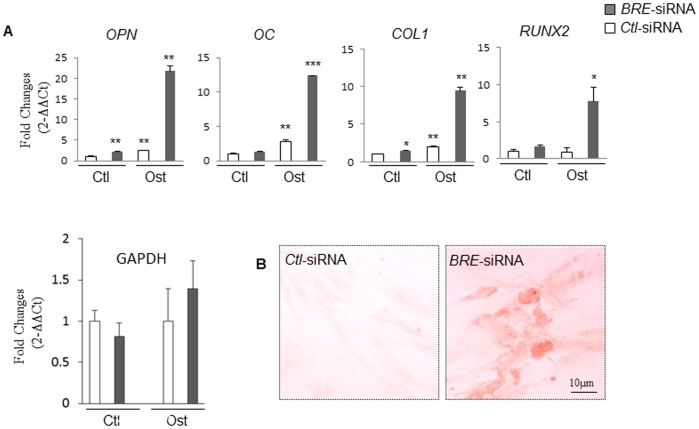
Silencing *BRE* expression accelerated osteogenic induction (requiring 3 days instead of 3 weeks). (A) RT-qPCR showing osteopontin (*OPN*), osteocalcin (*OC*), collagen I (*COL1*) and *RUNX2* gene expression patterns were strongly up-regulated in *BRE*-silenced HUCPV cells compared with cells treated with *Ctl-siRNAs*. All transfected cells were induced in osteogenic medium (ost) for 3-day. As a negative control, the *BRE*-siRNA and *Ctl-siRNAs* transfected cells were maintained in control medium (Ctl) 3-day for comparison. Gene expression was normalized against GAPDH. As a control, the stability of GAPDH during osteogenic differentiation was also investigated using GUSB for normalization. The results revealed that GAPDH expression did not alter significantly during differentiation – supporting our use of GAPDH as an internal control. (B) Alizarin red S staining revealed the presence of calcified bone matrix in the *BRE*-silenced HUCPV cells but not in control cultures. Both transfected cells were induced with osteogenic medium for 3-day. N = 3 independent experiments.

In this study, HUCPV cells were transfected with *BRE*-siRNA or *Ctl-siRNAs* prior to exposure to osteogenic inducing medium for 3 days. QRT-PCR analysis of these cultures revealed that there were significant increase in osteopontin (*OPN*), osteocalcin (*OC*), collagen I (*COL1*) and *RUNX2* expression in the induced *BRE*-silenced cells compared with induced cells transfected with *Ctl-siRNAs* (Figure 7A). Correspondingly, Alizarin red S staining revealed that the induced *BRE*-silenced HUCPV cultures contained calcified bone matrix whereas no staining was found in the control cultures (Figure 7B). Normally, 3 weeks were required to induce HUCPV cells to undergo osteogenesis in osteogenic inducing medium. Our results revealed that silencing *BRE* expression significantly accelerated this response as evident by the HUCPV cells expressing osteogenic gene markers and calcified matrix within 3 days of osteogenic induction.

**Figure 8:**
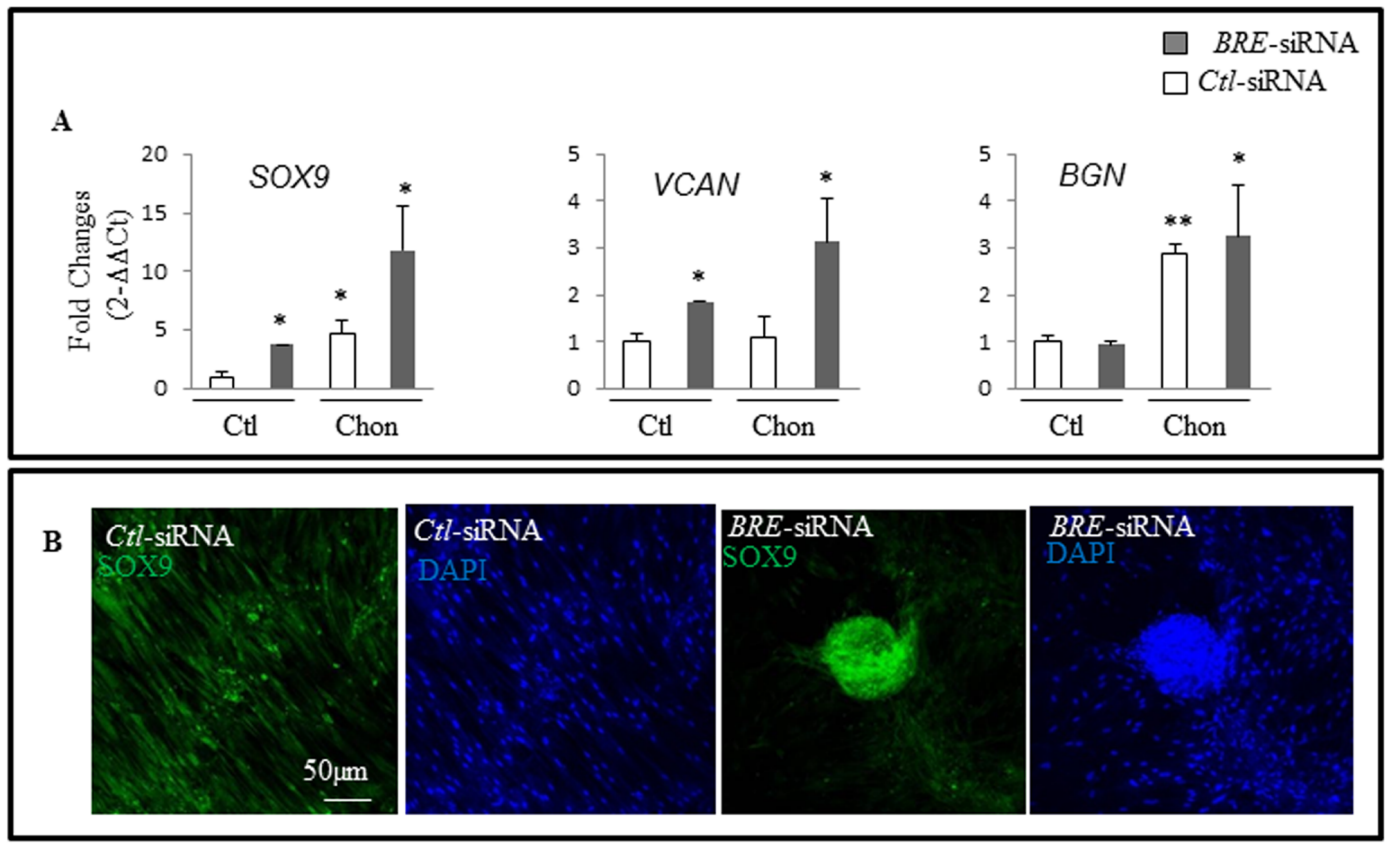
Silencing *BRE* expression accelerated chondrogenic (requiring 10 days instead of 4 weeks) induction. (A) RT-qPCR showing *SOX9*, versican (*VCAN*) and biglycan (*BGN*) expression patterns were strongly up-regulated in *BRE*-silenced HUCPV cells compared with control cells. All transfected cells were induced in chondrogenic medium (Chon) for 10-days. The transfected cells were also maintained in control medium (Ctl) for 10-days as a negative control. (B) Immunofluorescence staining confirmed that SOX9 was expressed by *BRE*-silenced HUCPV cells as compared with control cells. Both transfected cells were induced in chondrogenic medium for 10-days. The nuclei were counterstained with DAPI. (A, C). The statistics of P values were determined by t-test; *p<0.05, **p<0.01 and ***p<0.001 were considered significantly different. N = 3 independent experiments.

The same approach was used to assay whether chondrogenesis could be speeded up by transfecting HUCPV cells with *BRE*-siRNA prior to exposure to chondrogenic inducing medium. RT-qPCR analysis revealed that the *BRE*-silenced HUCPV cells strongly expressed chondrogenic marker *SOX9* and extracellular matrix proteoglycan versican (*VCAN*) after 10 days incubation in chondrogenic inducing medium, as compared with cells transfected with *Ctl-siRNAs* (Figure 8A). However, there was no significant up-regulation of biglycan (*BGN*), a small leucine-rich proteoglycan found in extracellular matrix (Figure 8A). Immunofluorescence staining confirmed that *BRE*-silenced HUCPV cells strongly expressed SOX9 after 10 days induction but not by HUCPV cells transfected with the *Ctl-siRNAs* (Figure 8B). Hence, it appears that silencing *BRE* expression in HUCPV cells could greatly speed up their response to chondrogenic inducing medium – where normally it would require 4 weeks instead of 10 days.

### Microarray analysis of *BRE*-silenced HUCPV cells

**Figure 9:**
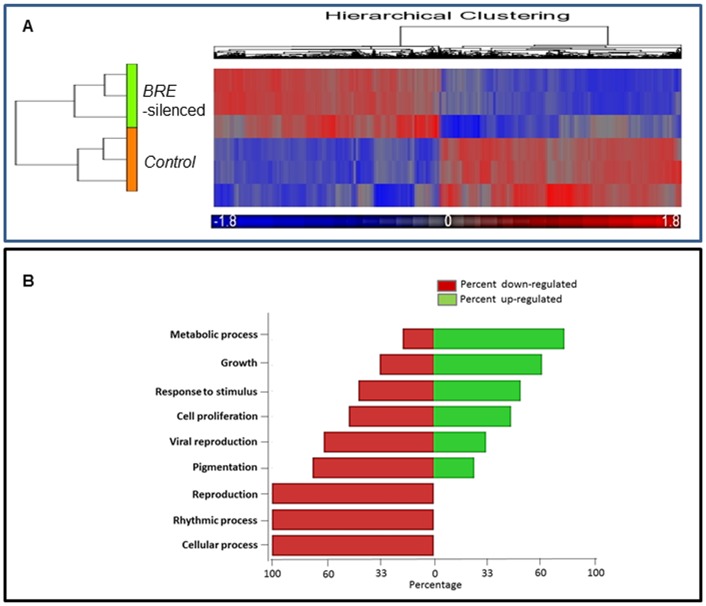
Microarray analyses HUCPV cells transfected with *BRE-siRNAs* and *Ctl-siRNAs*. (A) Illustrating the hierarchical clustering of genes that were differentially expressed. The gene expression profiles revealed that 1301 genes were significantly up-regulated (red) and 1433 genes were significantly down-regulated (blue) in *BRE*-silenced cells compared with control cells. Gene expression with ±2 fold change and the statistics of P values were determined by t-test; p<0.05 was considered significantly different. N = 3 independent experiments. (B) A forest plot showing the gene ontology enrichment of microarray results categorized according to biological processes.

Since silencing *BRE* expression could accelerate induced chondrogenic and osteogenic differentiation, we postulated that BRE might play an important role in regulating stemness and differentiation in HUCPV cells. In this context, we performed microarray expression profiling to ascertain the global changes in gene expression following *BRE*-silencing. HUCPV cell cultures were transfected twice with either *BRE*- or *Ctl-siRNAs* over a 24-hour interval. The transcriptome of *BRE*-silenced cells were then compared with cells transfected with *Ctl-siRNAs*. Only genes whose expression differed by at least 1.4-fold with a P value less than 5% were considered significant and deemed genes that are differentially expressed. We determined that there were 1301 genes up-regulated and 1433 genes down-regulated in *BRE*-silenced cells compared with the control cells. Hierarchical clustering performed on differentially expressed genes are shown in Figure 9A. These genes were enriched by gene ontology according to biological processes (Figures 9B). Cell proliferation, growth, and metabolic processes categories were the top three enriched scores. To specifically elucidate how *BRE*-silencing modulates stem cell function, gene sets that were significantly associated with developmental processes were investigated.

### BRE and Stemness factors

We first analyzed a panel of known pluripotent- and differentiation-related genes in detail [25], [26], [27], [28], [29], [30]. In response to *BRE*-silencing, we observed several pluripotent-related and stemness genes were down-regulated when compared with control HUCPV cells. Amongst these genes include *OCT4*, *AHCTF1*, *CDC2*, *COBRA1*, *FGF5*, *FOXO1A*, *LIFR*, *NOG* and *REST* (Figure S1A). RT-qPCR was performed to verify some of the down-regulated genes that we found - especially *OCT4*, as well as the other three key “Yamanaka factors” genes [31] (Figure S1B).

### BRE and Epigenetic regulation

Expressions of polycomb complex genes, which are key epigenetic regulators, were also found to be altered by *BRE*-silencing. There are two main Polycomb-repressive complexes (PRCs): PRC1 and PRC2 which are both capable of repressing hundreds of genes in mESCs. Components of the *PRC1* and *PRC2* genes that we found down-regulated by *BRE*-silencing include *PHC3*, *SCML2*, *EZH2*, *SUZ12P* and *PHF19*; whereas, the genes that were up-regulated include *L3MBTL* and *JARID2* (Figures S2A and B). Other genes involved in chromatin modifications, such as DNA methylation and histone modifications, were also altered in response to *BRE*-silencing. In our study, the expression of *HDAC9* was up-regulated following *BRE*-silencing. In addition, non-histone chromatin associated genes *CHAF1A*, *CHAF1B*, and *POLE2* were all down-regulated in response to *BRE*-silencing.

### BRE and TGF-β/BMP signaling

TGF-β/BMP signaling pathway has been extensively reported to play a crucial role in maintaining hESCs and hMSCs in an undifferentiated state and also in suppressing cell differentiation [32], [33], [34], [35], [36], [37]. Consistent with these reports, we have found that components of this signaling pathway were down-regulated by *BRE*-silencing. Specifically, *SMAD6*, *SMAD8*, and their downstream target genes *ID1*, *ID2*, *ID2B*, and *ID3* were all down-regulated (Figure S3A–C).

### BRE and FGF signaling

FGF signaling is essential for preventing hESCs differentiation and in maintaining hMSCs undifferentiated during their proliferative state [36], [38], [39], [40]. We observed that *FGF2*, *FGF9*, *FGF10*, *FGF21*, *FGFBP1*, *FGFR2* and *FGFR3* expressions were all down-regulated as a consequence of silencing *BRE* expression (Figure S4A and B).

### BRE and chemokines

The effects of *BRE*-silencing on chemokines and cytokines expression by HUCPV cells were investigated. The chemokines that were up-regulated include *CCL3*, *CXCL3*, *CXCL5*, *CXCL10*, *CXCL11*, and *CXCL12* (Figure S5A and B). A number of cytokines and receptors such as *TNFSF9*, *TNFSF15*, *TNFSF18*, *TNFRSF19*, *TNFRSF9*, *GDF5*, *IL1F9*, *IL7*, *IL15*, *IL1RAP*, *IL1RAPL2*, *IL6R*, *IL15RA*, *IL21R*, *IL31R*, *PDGFD* and *VEGF* were also up-regulated; whereas other cytokines such as *IFNA1*, *IFNA2*, *IFNA4*, *IFNA5*, *IFNA13*, *IFNA14*, *TNFAIP8*, *IL11*, *IL16*, *IL20*, *HTR2A*, *IFNA*, *EGF*, and *MPL* were down-regulated (Figure S6A–E).

### BRE and Homeobox (HOX) transcription factors

*HOX* encoded proteins are homeodomain-containing transcription factors that are important regulators of cell proliferation, self-renewal and differentiation in hematopoietic stem cells [41], [42], [43]. In response to *BRE*-silencing, the expression of *HOXB6*, *HOXB9*, and *HOXD4* were up-regulated while the expression of *HOXA2*, *HOXA3*, *HOXA10*, *HOXA11*, *HOXA13*, and *HOXB1* were down-regulated (Figure S7).

### *BRE*-silencing alters the proteome of HUCPV cells

We have also examined the proteome of *BRE*-silenced HUCPV cells and compared it with the proteome of HUCPV cells transfected with *Ctl-siRNAs*. The Protein lysates from both specimens were first resolved by 2-DE. Proteins that were determined to be differentially expressed were then identified by MALDI-TOF mass spectrometry. Seventy differentially expressed protein spots were detected. There were 19 protein spots that were up-regulated while 51 protein spots were down-regulated as a consequence of *BRE*-silencing. These proteins were categorized into their role in terms of molecular functions which was achieved by using the Database for Annotation, Visualization and Integrated Discovery (DAVID) program (Table S2) [44], [45]. Functional annotation clustering pointed to alterations in cytoskeletal protein binding in response to *BRE*-silencing in HUCPV cells. Furthermore, *BRE*-silencing response proteins appear to be involved in bindings of DNA, RNA, ATP and unfolded protein. In addition, the differentially expressed proteins have been annotated to regulate enzymatic activities such as isomerase and oxidoreductase activities.

### *BRE*-silencing alters expression of cytoskeletal binding proteins

**Figure 10:**
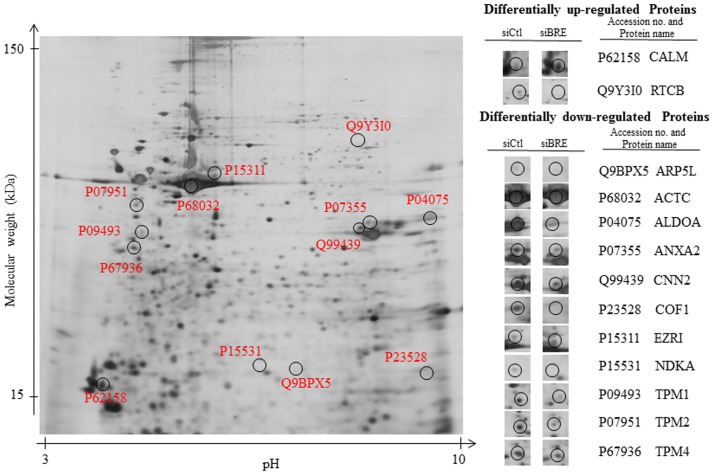
Comparative proteomics analysis of differentially expressed proteins in BRE silenced HUCPV cells. A representative silver stained 2-DE gel of total protein extracted from HUCPV cells that had been transfected with *BRE*-siRNA. When compared with control 2-DE gels, the ESI-MS/MS analysis identified proteins that were up- and down-regulated as a result of silencing *BRE*. Cytoskeletal binding proteins were especially affected. N = 3 independent experiments.

In the comparative proteomic study, *BRE*-silencing resulted in decreased expression of actin and tropomyosin proteins. Expressions of several actin modifying proteins, which are regulated by Rho-GTPases, were also found to be depleted in response to *BRE*-silencing [46]. These actin modifying proteins include annexin A2 (ANXA2), actin-related protein 2/3 (Arp2/3) complex subunit 5-like protein (ARP5L), fructose-bisphosphate aldolase A (ALDOA), calponin-2 (CNN2), cofilin-1 (COF1), and ezrin (EZRI) (Figure 10 and Table S3). In contrast, expressions of calmodulin (CALM) and chromosome 22 open reading frame 28 (RTCB) were up-regulated in response to *BRE*-silencing.

**Figure 11:**
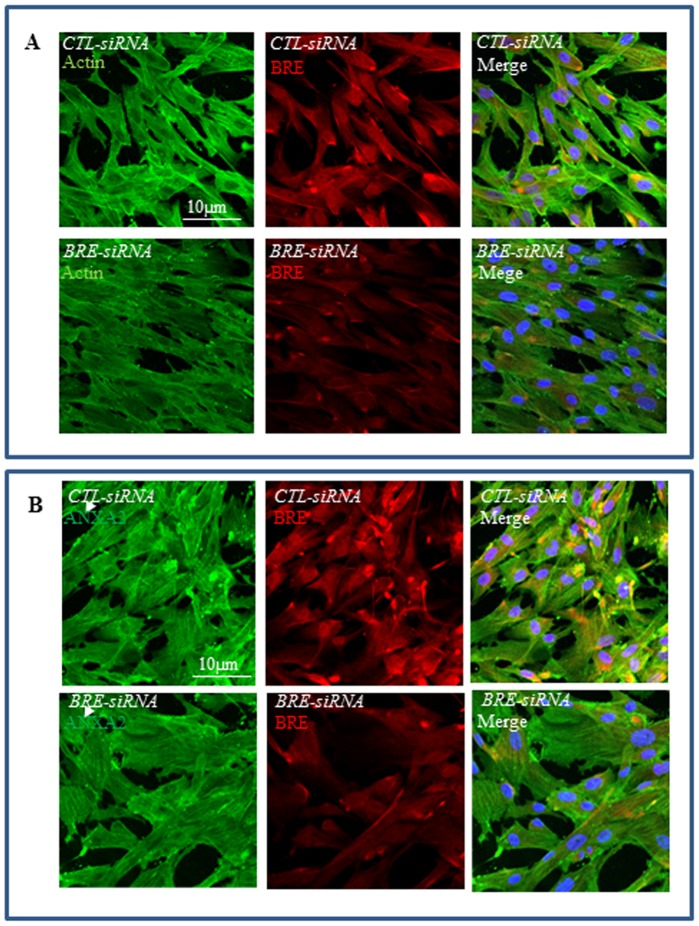
Immunofluorescence staining of Actin, BRE and ANXA2 expression in BRE-silenced HUCPV cells. (A) Showing actin (green) and BRE (red) expressions were inhibited in *BRE*-silenced HUCPV cells compared with the control. Arrows indicate where actin and BRE were co-localized. (B) Showing ANXA2 (green) expression was down-regulated following *BRE*-silencing. N = 3 independent experiments.

**Figure 12:**
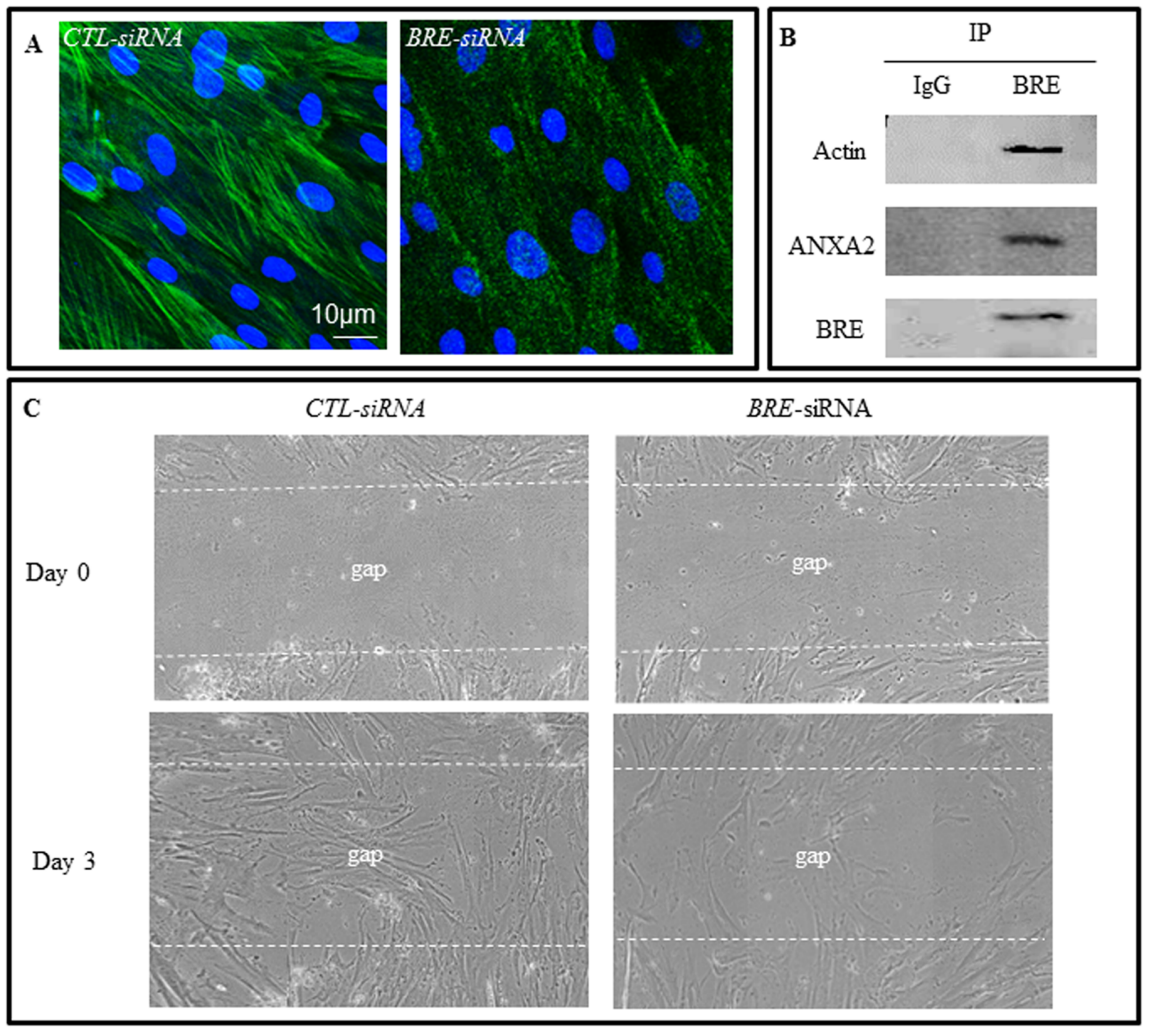
*BRE*-silencing alters expression of cytoskeletal binding proteins and cell migration. (A) Phalloidin staining revealed F-actin were highly aligned and structured in HUCPV cells but became diffusely distributed in *BRE*-silenced cells. (B) Immunoprecipitation of endogenous BRE was performed on HUCPV cell lysates using BRE monoclonal antibodies. The resultant immunoprecipitates were separated by SDS-PAGE and Western blot analysis performed using antibodies against BRE, actin and ANXA2. The results revealed that BRE can directly bind actin and ANXA2. (C) Scratch cell migration assay demonstrated that *BRE*-silencing inhibited HUCPV cell migration. The dotted white lines indicate the extent of the gap created which were completely invaded by HUCPV cells in the control cultures, after 3 days. In contrast, the gap was only partially invaded in *BRE*-silenced cultures at day-3. N = 3 independent experiments.

We selected actin and ANXA2 for further investigation. Immunofluorescence staining microscopy confirmed our proteomic results that actin and ANXA2 expressions were reduced in *BRE*-silenced HUCPV cells (Figures 11A and B). Furthermore, in co-immunofluorescence staining of cultured HUCPV cells, we found actin and BRE were was co-localized in the cytoplasm. Likewise, ANXA2 was also co-localized with BRE. In addition, phalloidin staining revealed that the highly organized F-actin structures seen in control HUCPV cells were totally disassembled in *BRE*-silenced HUCPV cells (Figure 12A). We also observed striation patterns when BRE antibody was used in immunofluorescence studies. Therefore, we performed immunoprecipitation study to determine whether actin and ANXA2 can directly bind endogenous BRE. We used monoclonal BRE specific antibody to pull down the proteins in cell lysates. We determined that BRE can directly associate with actin and ANXA2 (Figure 12B). Since actin and ANXA2 are also involved in regulating cell motility, we investigated whether cell migration was altered in *BRE*-silenced HUCPV cells using a Scratch assay. We observed that *BRE*-silenced cells migrated at a slower rate than cells transfected with *Ctl-siRNAs* in the Scratch assay. Approximately 40±8.6% of the area of the gap was filled up with *BRE*-silenced cells compared with 96±10% for *Ctl-siRNAs* treated cells (Figure 12C). The experiment was repeated in triplicate. The statistical difference of P values were determined by t-test; p<0.05, was considered significantly different.

### *BRE*-silencing alters expression of stemness-related proteins

We have closely examined the proteomic results for proteins that maintain stemness and regulate stem cell differentiation. The results revealed that calreticulin (CALR), far upstream element-binding protein 1 (FUBP1) and pyruvate kinase isozymes M1/M2 (PKM2) expression were all down-regulated when *BRE* was silenced in HUCPV cells (Table S4).

## Discussion

In this study, we demonstrated that HUCPV cells transient transfected with *BRE*-siRNA could accelerate osteogenic and chondrogenic differentiation when they are cultured in the appropriate inducing medium. Normally, osteogenic induction of HUCPV cells requires approximately three weeks to take place while chondrogenic induction requires even longer, four weeks. In contrast, when BRE expression was silenced, HUCPV cell differentiation was greatly speeded up – 3 days instead of 3 weeks were required for osteogenic induction and 10 days instead of 4 weeks for chondrogenic induction. Previous studies have demonstrated that using BMP-2 and dexamethasone, it was possible to rapidly induce rat bone marrow stromal cells to become osteoblasts, requiring only 8 days [47]. Presently, we revealed that silencing *BRE* expression prior to osteogenic induction could enhance osteogenesis even faster than by adding exogenous BMP-2. This observation is particularly important in the field of regenerative medicine, especially in the context of non-union bone and cartilage fractures when rapid repair is highly desirable to prevent later complications.

To elucidate the function of BRE in HUCPV cells, we employed microarray analysis. We identified a number of BRE targeted genes in HUCPV cells that have important roles in maintaining stemness. We grouped these genes into different categories. In *BRE*-silenced HUCPV cells, we observed *OCT4*, *FGF5*, and *FOXO1A* expression was down-regulated and these transcriptional genes are essential for maintaining pluripotency in ES cells. Especially OCT4, which is part of the core transcriptional regulatory circuit that maintains stem cell pluripotency by activating self-renewal genes and suppressing genes that promote differentiation [26], [27]. This finding is further collaborated by the fact that *HMGB2*, a co-activator of OCT4 activity, was also correspondingly down-regulated following *BRE* silencing [48]. In addition, we also established that pluripotent epiblast marker *FGF5* and the recently identified essential hESCs pluripotent gene *FOXO1A* were also inhibited in response to *BRE*-silencing [49], [50], [51], [52]. Expressions of epigenetic and chromatin modifier genes were also affected - with *HDAC9* expressions enhanced following *BRE*-silencing. Histone deactylase genes perform diverse stem cell function [53]. In agreement with our observation where *BRE*-silencing promoted osteogenenic differentiation, it has been shown that HDAC9 played a crucial role in enhancing hMSCs osteogenesis [54]. These results indicate that *BRE*-silencing participates in modulating the genes involved in maintaining stemness and chromatin modification.

We also observed expression of genes encoding for components of the TGF-β/BMP, and FGF signaling pathways repressed in response to *BRE*-silencing. These pathways work to promote self-renewal and suppress differentiation [34], [35], [36], [37]. *SMAD6*, *SMAD8*, *ID1*, *ID2*, and *ID3*, all of them were negatively regulated by *BRE*-silencing, have been shown to inhibit osteogenic differentiation in MSCs [55], [56]. FGF2 has also been identified as one of the self-renewal factors which can sustain the growth of hESCs and hMSCs on a feeder-independent and serum-free condition [57], [58]. Moreover, FGF9 has been implicated in promoting survival of germ cells in fetal testis and it is a highly specific ligand for FGF receptor 2 and 3 which maintain pluripotency in mouse germ cells [59], [60], [61]. It has been reported that FGF signaling through FGFR2 inhibits mouse osteoblast differentiation [62]. A few studies have indicated that FGF2 could also inhibit multi-lineage MSC differentiations, while other studies have suggested FGF2 was a positive regulator of MSCs differentiation [63], [64], [65], [66]. The contradictory effects may be dependent on the developmental status of the stem cells as it was reported that FGF2 stimulates growth of immature osteoblast progenitors but induces osteogenic differentiation of more mature precursors [67]. Our study suggests that FGF signaling inhibits HUCPV cells differentiation. *BRE*-silencing negatively regulated genes such as *FGF2*, *FGF9*, *FGFR2* and *FGFR3*; thereby permitting HUCPV cells to differentiate into osteoblasts more rapidly when induced.

*HOX* transcription factors are important regulators of cell proliferation, self-renewal and differentiation in hematopoietic stem cells (HSCs) [41], [43]. Overexpression studies have reported that HOXB4 could enhance mHSCs expansion and mESCs differentiation [68], [69]. Furthermore, overexpression of HOXB4 up-regulates the transcriptional expression of *BRE* in mHSCs [68]. In our microarray study, however, we did not observe a significant change of *HOXB4* expression in response to *BRE*-silencing. This observation implied that HOXB4 regulates the expression of *BRE* but not vice versa. Therefore, it will be interesting to establish whether HOXB4 overexpression can up-regulate BRE expression in mesenchymal stem cells or whether the protein regulation is lineage specific.

*BRE*-silencing resulted in the alteration of a number of chemokines. Amongst the chemokines that were up-regulated includes, *TNFSF15*, *TNFRSF9*, *GDF5* and *IL7*. These chemokines have been implicated in their ability to promote osteogenesis of various types of progenitor cells [70], [71], [72], [73]. Other cytokines that have been enhanced in response to *BRE*-silencing were *TNFRSF9*, *IL7*, *IL15RA* and *IL21R* which are up-regulated as hESCs differentiate into embryoid bodies [74]. Furthermore, CXCL3, CXCL10 and TNFSF9, which were positively regulated by *BRE*-silencing, have been classified as inhibitory factors in a study of intercellular network which modulated hematopoietic stem cell fate non-autonomously [75]. Therefore, *BRE*-silencing modulates the expression of cytokines which provided a favorable environment for differentiation to progress.

We also employed comparative proteomics approach to elucidate all the proteins that are differentially expressed after *BRE*-silencing in HUCPV cells which provided valuable insight on BRE targeted post-translational proteins to complement the microarray study. Functional annotation clustering analysis revealed that BRE function was associated with the organization of the cytoskeleton. *BRE*-silencing resulted in decreased presence of actin, ANXA2 and other cytoskeletal binding proteins. We also demonstrated that actin and ANXA2 could be immunoprecipitated with BRE. ANXA2 has been shown to bind to F-actin and plays a critical role in Rho/ROCK signaling pathway [76], [77]. ANXA2 along with other actin-binding proteins are capable of regulating actin dynamics, organization and turnover in migratory cells [76], [78], [79], [80], [81], [82]. Actins, together with intermediate filaments and microtubules, play an important role in cytoskeletal organization and response to external mechanical stimuli that affect cell differentiation [83], [84], [85]. During differentiation, the cell morphology is almost always altered in respond to integrins, cadherins and cytoskeletal proteins being differentially expressed [86]. When MSCs undergo osteogenesis, the cells become flatten and spread out; while the cells acquire a spherical shape when they undergo chondrogenesis [87], [88]. It has been demonstrated when actin is disrupted by cytochalasin B (an inhibitor of actin polymerization), chondrogenesis is greatly enhanced in ESCs and MSCs. In contrast, chondrogenesis is hindered under conditions that favour actin assembly [89], [90], [91], [92]. In our study, we demonstrated that the structural integrity of F-actin became dissembled in *BRE*-silenced HUCPV cells while F-actin structures were very distinctive in cells transfected with *Ctl-siRNAs*. The reduction of actin protein in *BRE*-silenced cells might provide a favourable environment for differentiation to take place, which agrees with our experimental observation that *BRE*-silencing accelerated chondrogenic differentiation of HUCPV cells.

Because actin and ANXA2 play a role in cell migration, we investigated the effect of *BRE*-silencing on cell movement. The HUCPV cells showed reduced ability to migrate after *BRE*-silencing. The reduction is probably associated with the down-regulation of actin protein which affected the cytoskeleton to change dynamically during movement – since migration requires a synchronized interactions of actin and its interacting partners such as actin-binding proteins and integrins [93]. ANXA2 has also been implicated in the regulation of HSC binding to osteoblasts and homing to the bone marrow niche [94]. Further studies will be required to establish whether BRE has a role in cell homing.

*BRE*-silencing could also suppress CALR, FUBP1 and PKM2 expression. In mESCs, down-regulation of CALR resulted in reduction of calcium ion level to enhance adipogenic differentiation [95]. It has also been reported that CALR may be involved in the regulation of osteogenesis and chondrogenesis in MSCs. CALR expression is reduced in the early stage of osteoblast differentiation in MC-3T3-E1 cells. Overexpression of CALR inhibits both the basal and vitamin D-induced expression of osteocalcin and calcium ion accumulation in the extracellular matrix and mineralization of bone nodules in cultures [96]. These findings may explain our observation of why *BRE*-silencing resulted in enhanced osteogenic and chondrogenic differentiation in HUCPV cells. *BRE*-silencing also negatively regulates FUBP1 and PKM2. FUBP1 is present in undifferentiated cells but not in differentiated cells and regulates c-myc expression by binding to a single-stranded far-upstream element upstream of the c-myc promoter [97]. Previous report showed that down-regulation of FUBP1 and subsequent down-regulation of c-myc were needed for lung cell differentiation in mice [98]. This protein may act both as an activator and repressor of transcription. Likewise, PKM2 has been determined to stimulate Oct4-mediated transcriptional activation [99]. Oct4 and c-myc constitute are two of the Yamanaka transcription factors crucial for maintaining pluripotency of stem cells [31], [100]. *BRE*-silencing resulted in decreased expression of pluripotency factors and may explain our observation of enhanced osteogenic and chondrogenic differentiation in HUCPV cells. Further studies are required to examine the effect of *BRE*-silencing on other mesenchymal lineage differentiations.

**Figure 13:**
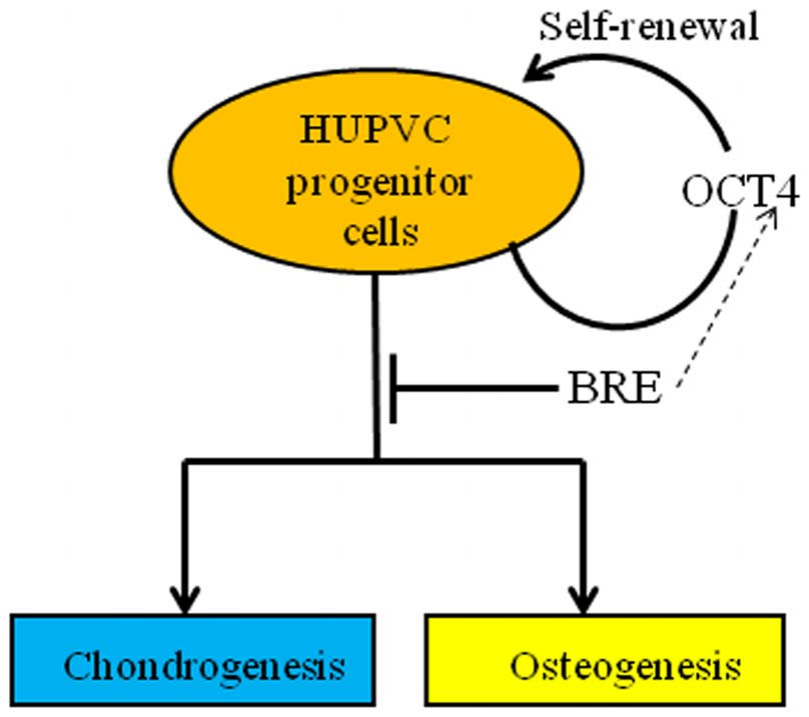
Propose model of BRE function in HUPVC cells. BRE is normally involved in inhibiting HUPVC cell differentiation and the maintenance of OCT4 expression. When BRE expression is silenced, OCT4 expression is partially inhibited and HUPVC cell differentiation is accelerated following osteogenic or chondrogenic induction.

In sum, our findings suggest a multifunctional role for BRE in maintaining stemness and the cytoskeletal architecture of HUCPV cells. In addition, BRE expression can be manipulated to accelerate induced chondrogenic and osteogenic differentiation in HUCPV cells (Figure 13).

## Supporting Information

Figure S1 **Microarray analyses of differentially expressed stemness-related genes in response to** ***BRE*** **-silencing.** (A) Heat map displaying the patterns of differentially expressed stemness genes in response to *BRE*-silencing. The red boxes indicate that gene signals that are higher than the background signal (grey boxes), whereas blue boxes indicate signals that are lower than background. (B) RT-qPCR was performed to confirm the microarray data, specifically genes associated with stemness. Gene expression was normalized to GAPDH. The statistics of P values were determined by t-test; *p<0.05, **p<0.01, ***p<0.001 were considered significantly different and #contrast with microarray results.
(TIF)

Figure S2 **Microarray analyses of differentially expressed epigenetic-related genes in response to** ***BRE*** **-silencing.** (A) Heat map displaying the patterns of differentially expressed epigenetic genes in response to *BRE*-silencing. (B) RT-qPCR was performed to confirm the microarray data, specifically genes associated with epigenetics. Gene expression was normalized to GAPDH. The statistics of P values were determined by t-test; *p<0.05, **p<0.01, ***p<0.001 were considered significantly different and #contrast with microarray results. N = 3 independent experiments.
(TIF)

Figure S3 **Microarray analyses of differentially expressed genes associated with TGF-β signalling in response to** ***BRE*** **-silencing.** (A) Heat map displaying the patterns of differentially expressed genes associated with TGF-β signalling in response to *BRE*-silencing. (B) RT-qPCR was performed to confirm the microarray data, specifically genes associated with TGF-β signalling. Gene expression was normalized to GAPDH. The statistics of P values were determined by t-test; *p<0.05, **p<0.01, ***p<0.001 were considered significantly different. N = 3 independent experiments. (C) Illustrating the cascade associated with the TGF-β signalling pathway (adapted from the KEGG database). Genes within the green boxes are significantly affected by BRE silencing while genes within the dark boxes are considered insignificantly affected.
(TIF)

Figure S4 **Microarray analyses of differentially expressed genes associated with FGF signalling in response to** ***BRE*** **-silencing.** (A) Heat map displaying the patterns of differentially expressed genes associated with FGF signalling in response to *BRE*-silencing. (B) RT-qPCR confirming the microarray data. Gene expression was normalized to GAPDH. The statistics of P values were determined by t-test; *p<0.05, **p<0.01, ***p<0.001 were considered significantly different. N = 3 independent experiments.
(TIF)

Figure S5 **Microarray analyses of differentially expressed chemokine genes in response to** ***BRE*** **-silencing.** (A) Heat map displaying the patterns of differentially expressed chemokine genes. (B) RT-qPCR confirming the microarray data. Gene expression was normalized to GAPDH. The statistics of P values were determined by t-test; *p<0.05, **p<0.01, ***p<0.001 were considered significantly different. N = 3 independent experiments.
(TIF)

Figure S6 **Microarray analyses of differentially expressed TNF family-related genes in response to** ***BRE*** **-silencing.** (A) Heat map displaying the patterns of differentially expressed TNF family-related and interleukin genes. (B) RT-qPCR confirming the microarray data. Gene expression was normalized to GAPDH. The statistics of P values were determined by t-test; *p<0.05, **p<0.01, ***p<0.001 were considered significantly different. N = 3 independent experiments.
(TIF)

Figure S7 **Microarray analyses of differentially expressed Hox genes in response to** ***BRE*** **-silencing.** (A) Heat map displaying the patterns of differentially expressed Hox genes. The red boxes indicate that the gene signal is 36 higher than the background signal (grey boxes); whereas blue boxes indicate the signal is lower than background.
(TIF)

Table S1 **The sequences of primers used in the RT-qPCR.**
(JPG)

Table S2 **The top 20 proteins identified by comparing** ***BRE*** **-silenced and control human umbilical cord perivascular cells, grouped based on molecular function using Gene Ontology tools.**
(JPG)

Table S3 **Cytoskeletal binding proteins differentially expressed in** ***BRE*** **-silenced human umbilical cord perivascular cells.**
(JPG)

Table S4 **Stemness-associated proteins differentially expressed in** ***BRE*** **-silenced human umbilical cord perivascular cells.**
(JPG)
