# Supplementary figures and images for: Silencing BRE Expression in Human Umbilical Cord Perivascular (HUCPV) Progenitor Cells Accelerates Osteogenic and Chondrogenic Differentiation
Source: PLoS One. 2013 Jul 23;8(7):e67896. doi: 10.1371/journal.pone.0067896 (PMC3720665; doi:10.1371/journal.pone.0067896)

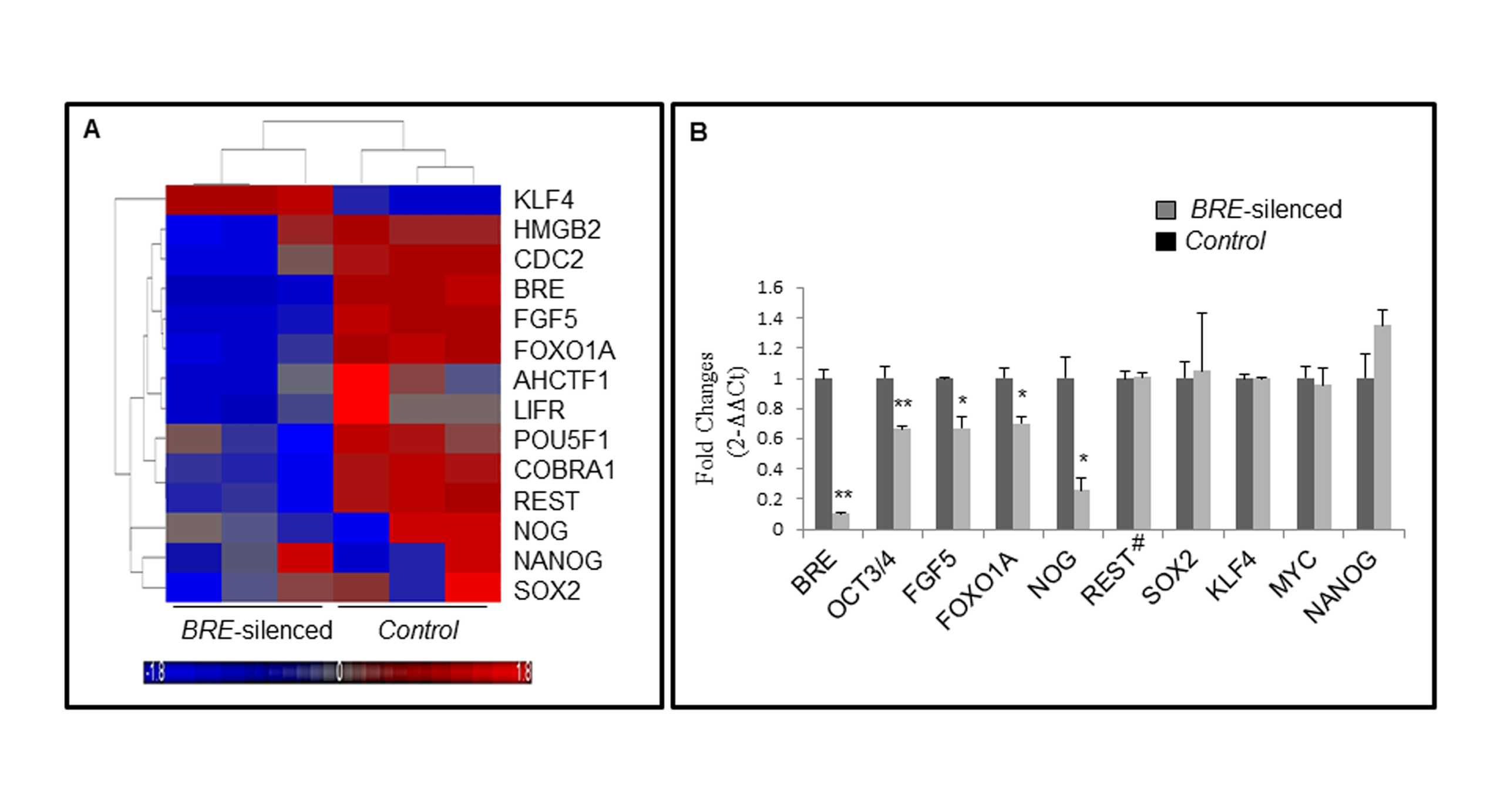

Supplement: Figure S1 — Microarray analyses of differentially expressed stemness-related genes in response to BRE -silencing. (A) Heat map displaying the patterns of differentially expressed stemness genes in response to BRE-silencing. The red boxes indicate that gene signals that are higher than the background signal (grey boxes), whereas blue boxes indicate signals that are lower than background. (B) RT-qPCR was performed to confirm the microarray data, specifically genes associated with stemness. Gene expression was normalized to GAPDH. The statistics of P values were determined by t-test; *p<0.05, **p<0.01, ***p<0.001 were considered significantly different and #contrast with microarray results. (TIF) [file pone.0067896.s001.tif]

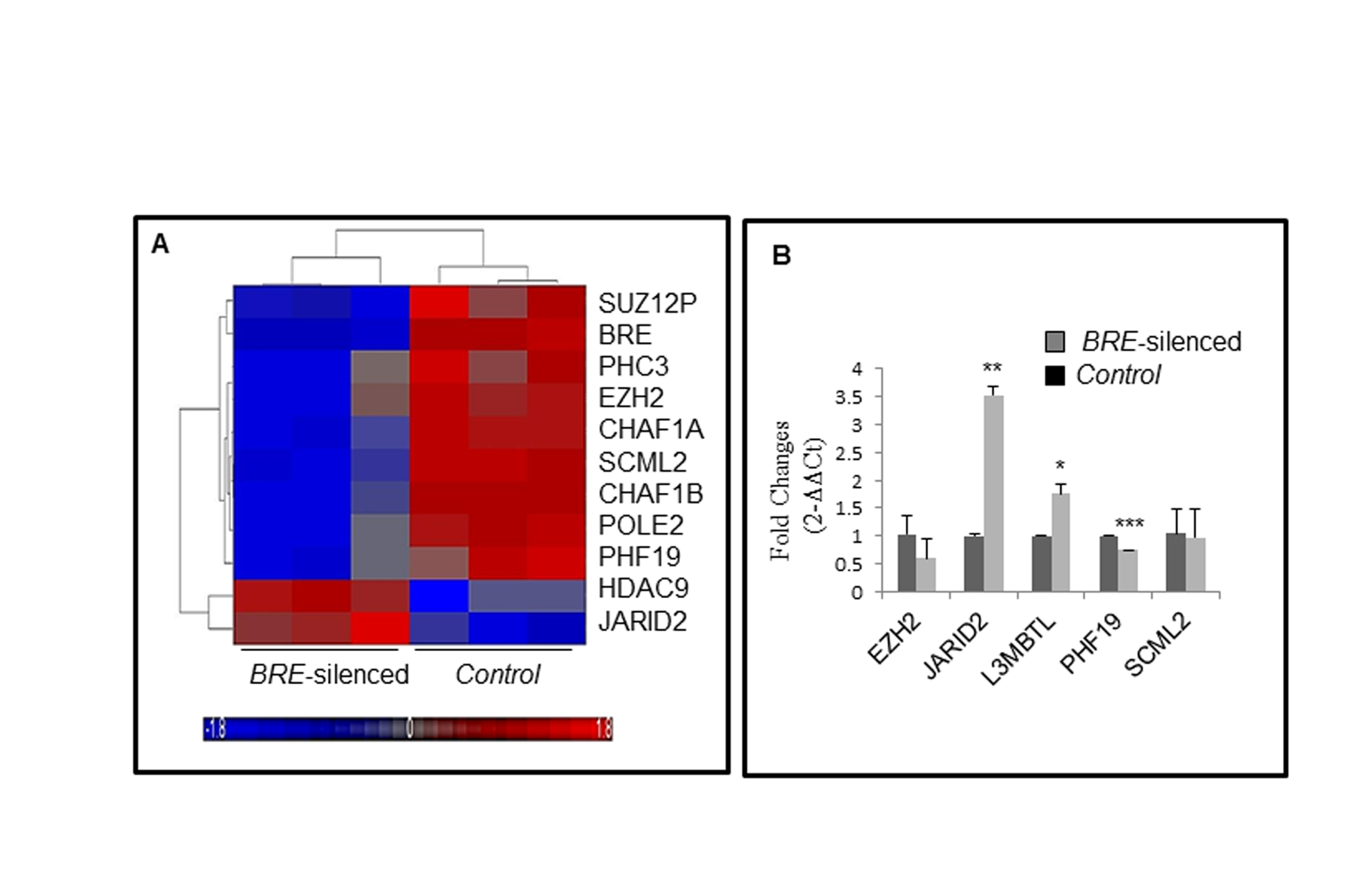

Supplement: Figure S2 — Microarray analyses of differentially expressed epigenetic-related genes in response to BRE -silencing. (A) Heat map displaying the patterns of differentially expressed epigenetic genes in response to BRE-silencing. (B) RT-qPCR was performed to confirm the microarray data, specifically genes associated with epigenetics. Gene expression was normalized to GAPDH. The statistics of P values were determined by t-test; *p<0.05, **p<0.01, ***p<0.001 were considered significantly different and #contrast with microarray results. N = 3 independent experiments. (TIF) [file pone.0067896.s002.tif]

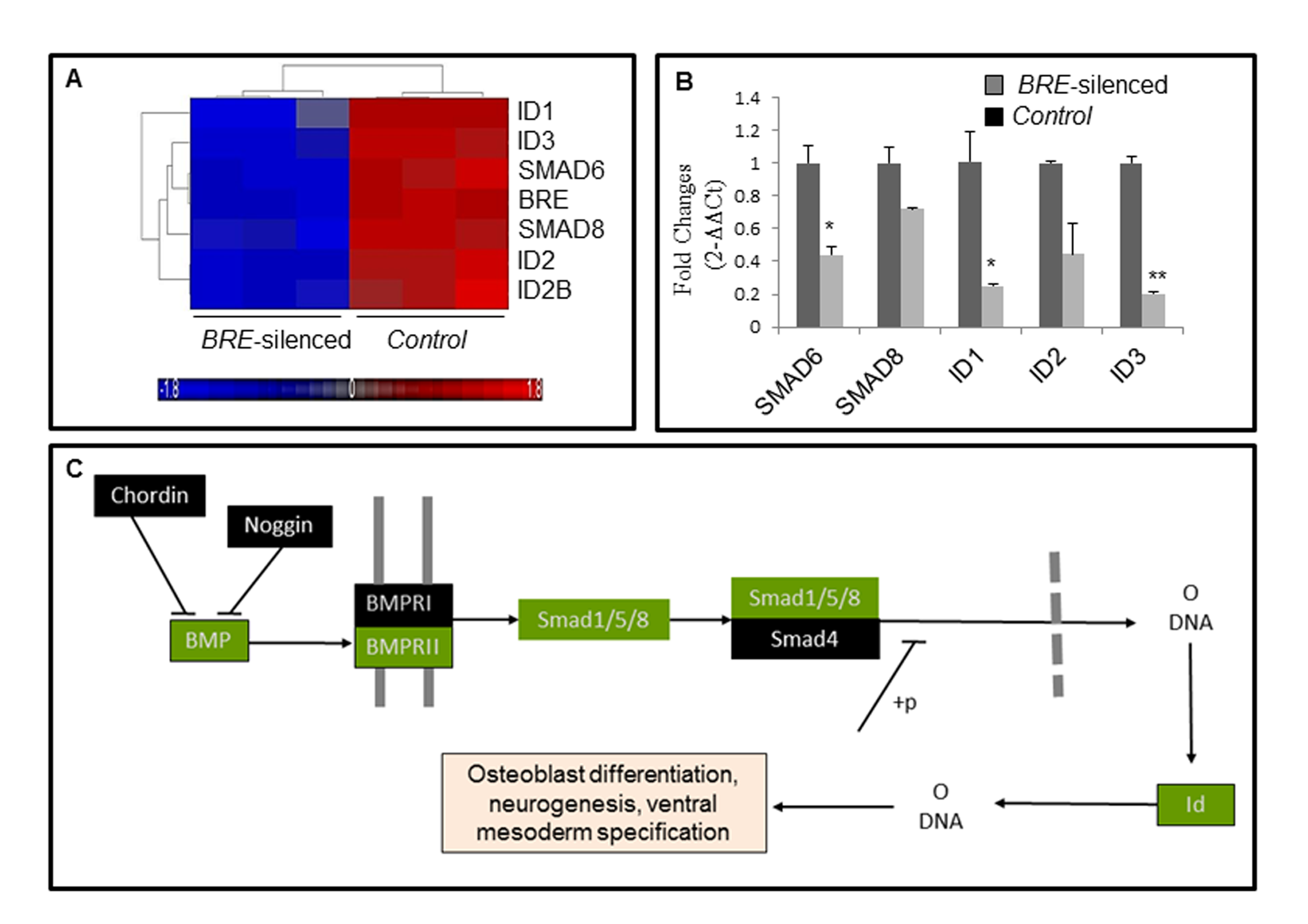

Supplement: Figure S3 — Microarray analyses of differentially expressed genes associated with TGF-β signalling in response to BRE -silencing. (A) Heat map displaying the patterns of differentially expressed genes associated with TGF-β signalling in response to BRE-silencing. (B) RT-qPCR was performed to confirm the microarray data, specifically genes associated with TGF-β signalling. Gene expression was normalized to GAPDH. The statistics of P values were determined by t-test; *p<0.05, **p<0.01, ***p<0.001 were considered significantly different. N = 3 independent experiments. (C) Illustrating the cascade associated with the TGF-β signalling pathway (adapted from the KEGG database). Genes within the green boxes are significantly affected by BRE silencing while genes within the dark boxes are considered insignificantly affected. (TIF) [file pone.0067896.s003.tif]

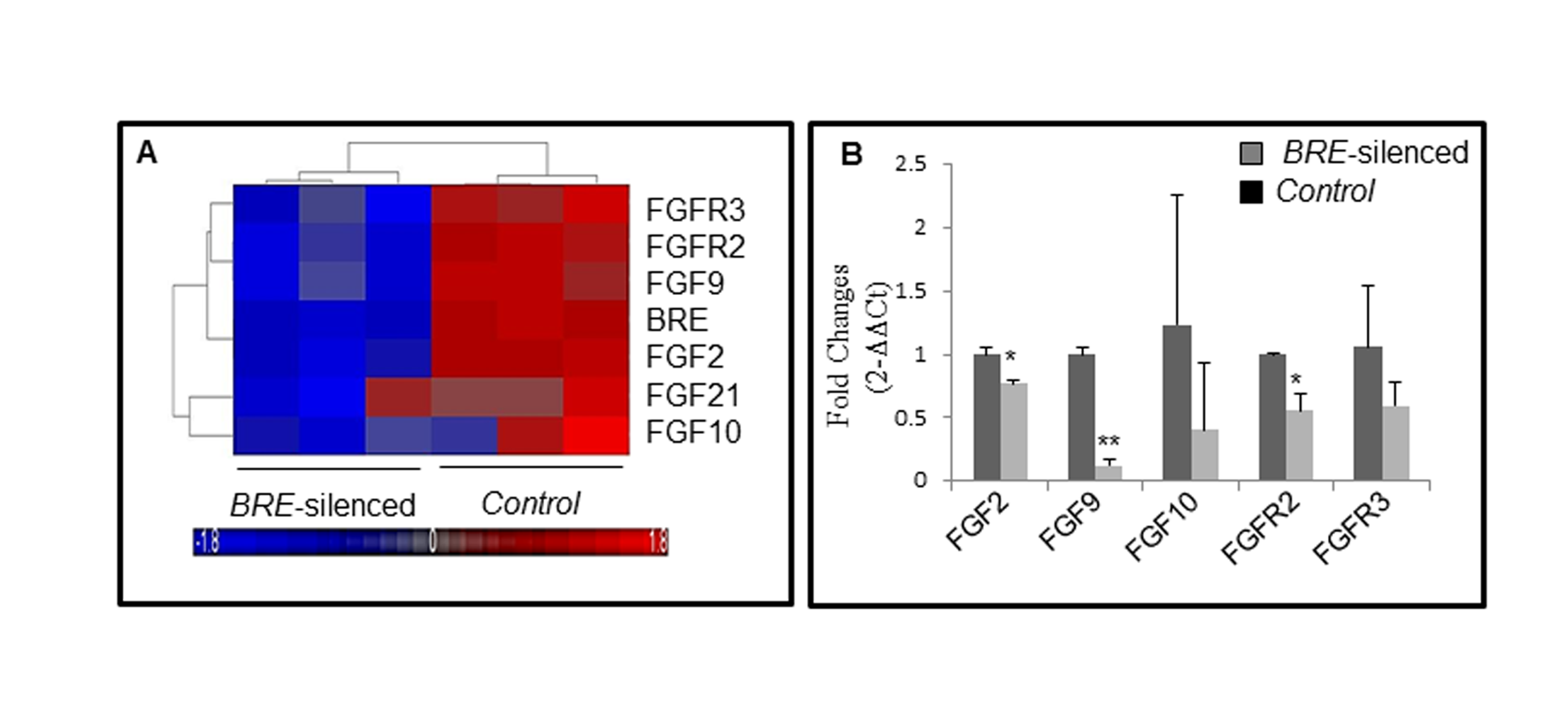

Supplement: Figure S4 — Microarray analyses of differentially expressed genes associated with FGF signalling in response to BRE -silencing. (A) Heat map displaying the patterns of differentially expressed genes associated with FGF signalling in response to BRE-silencing. (B) RT-qPCR confirming the microarray data. Gene expression was normalized to GAPDH. The statistics of P values were determined by t-test; *p<0.05, **p<0.01, ***p<0.001 were considered significantly different. N = 3 independent experiments. (TIF) [file pone.0067896.s004.tif]

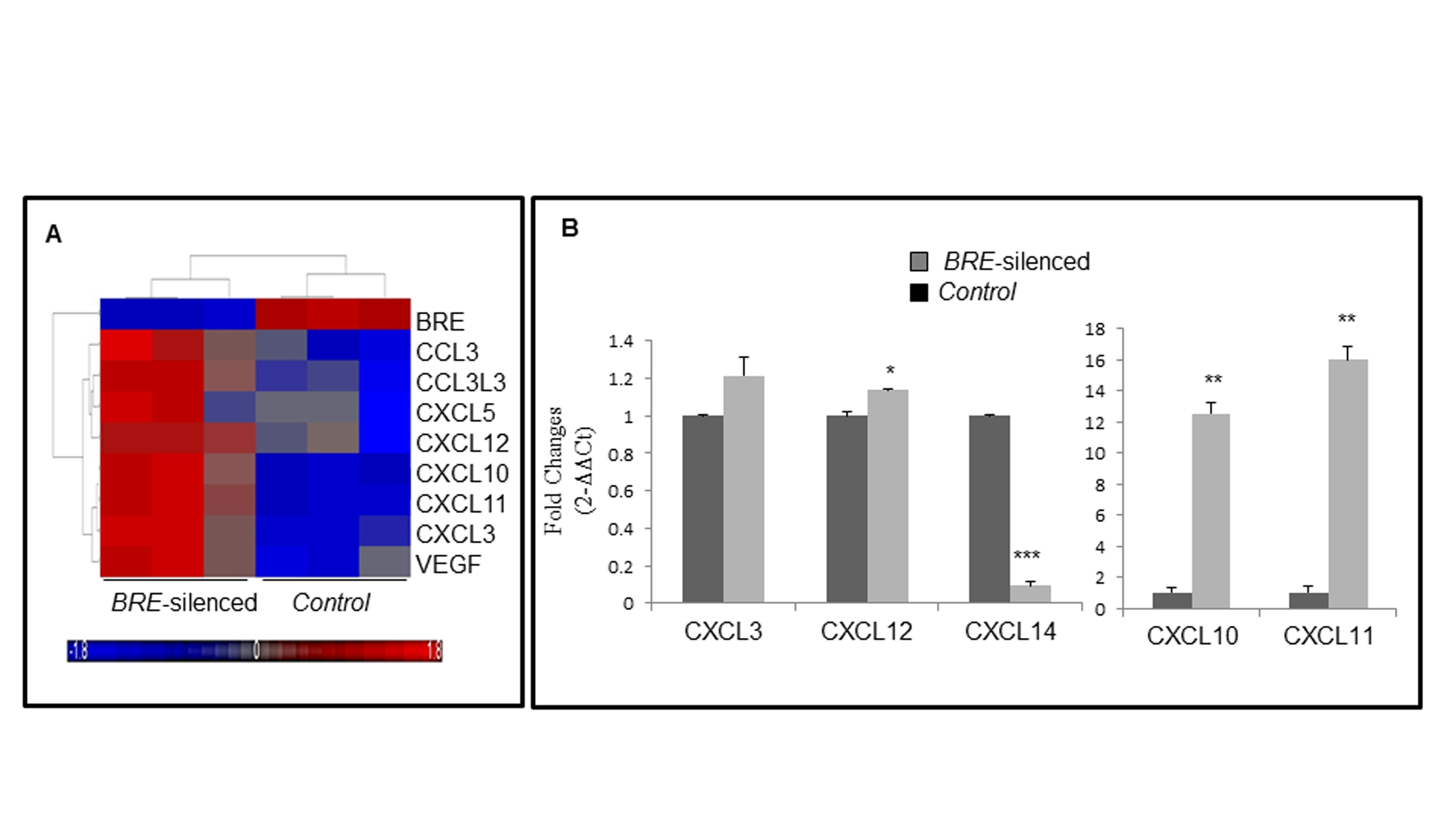

Supplement: Figure S5 — Microarray analyses of differentially expressed chemokine genes in response to BRE -silencing. (A) Heat map displaying the patterns of differentially expressed chemokine genes. (B) RT-qPCR confirming the microarray data. Gene expression was normalized to GAPDH. The statistics of P values were determined by t-test; *p<0.05, **p<0.01, ***p<0.001 were considered significantly different. N = 3 independent experiments. (TIF) [file pone.0067896.s005.tif]

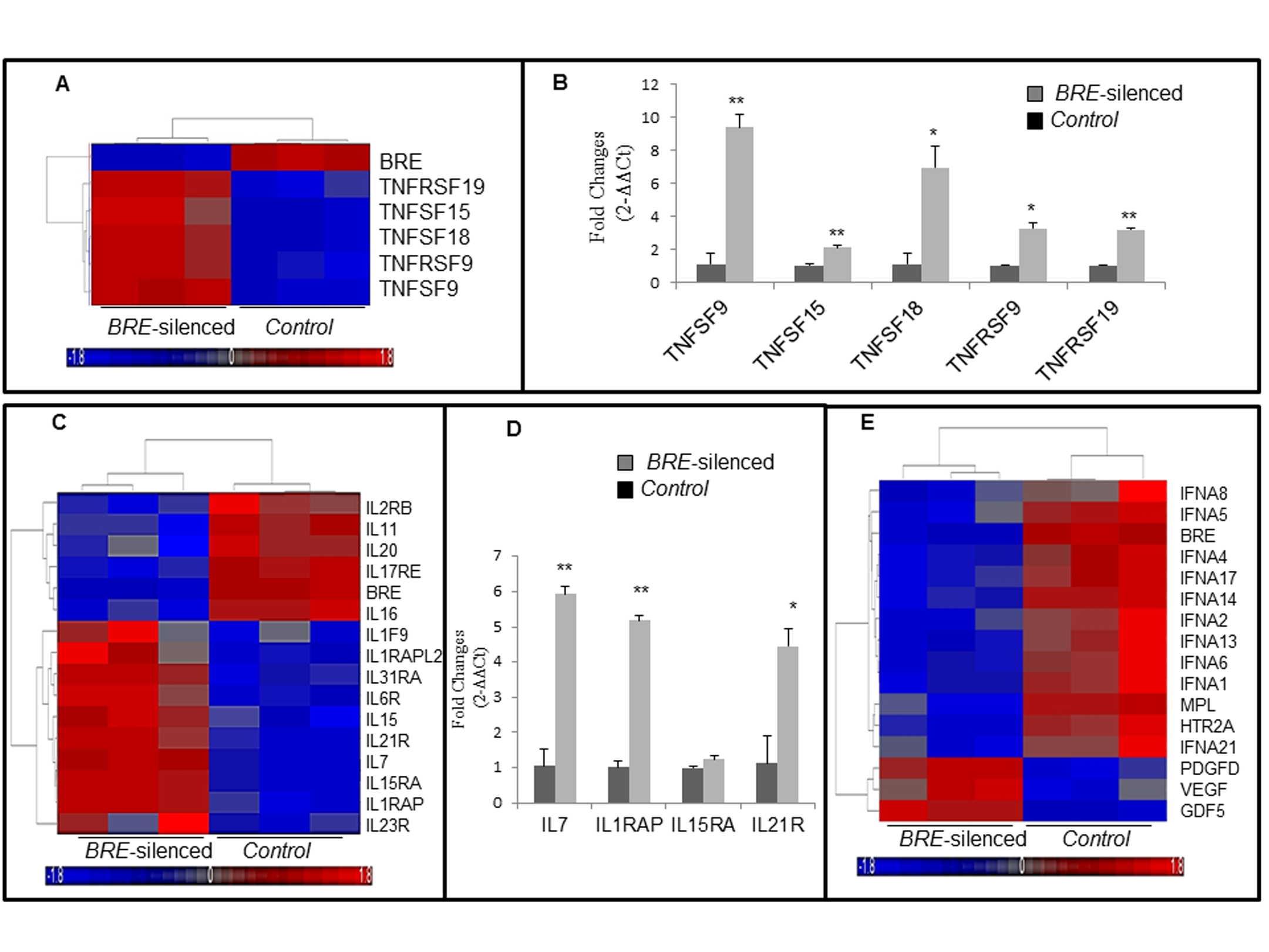

Supplement: Figure S6 — Microarray analyses of differentially expressed TNF family-related genes in response to BRE -silencing. (A) Heat map displaying the patterns of differentially expressed TNF family-related and interleukin genes. (B) RT-qPCR confirming the microarray data. Gene expression was normalized to GAPDH. The statistics of P values were determined by t-test; *p<0.05, **p<0.01, ***p<0.001 were considered significantly different. N = 3 independent experiments. (TIF) [file pone.0067896.s006.tif]

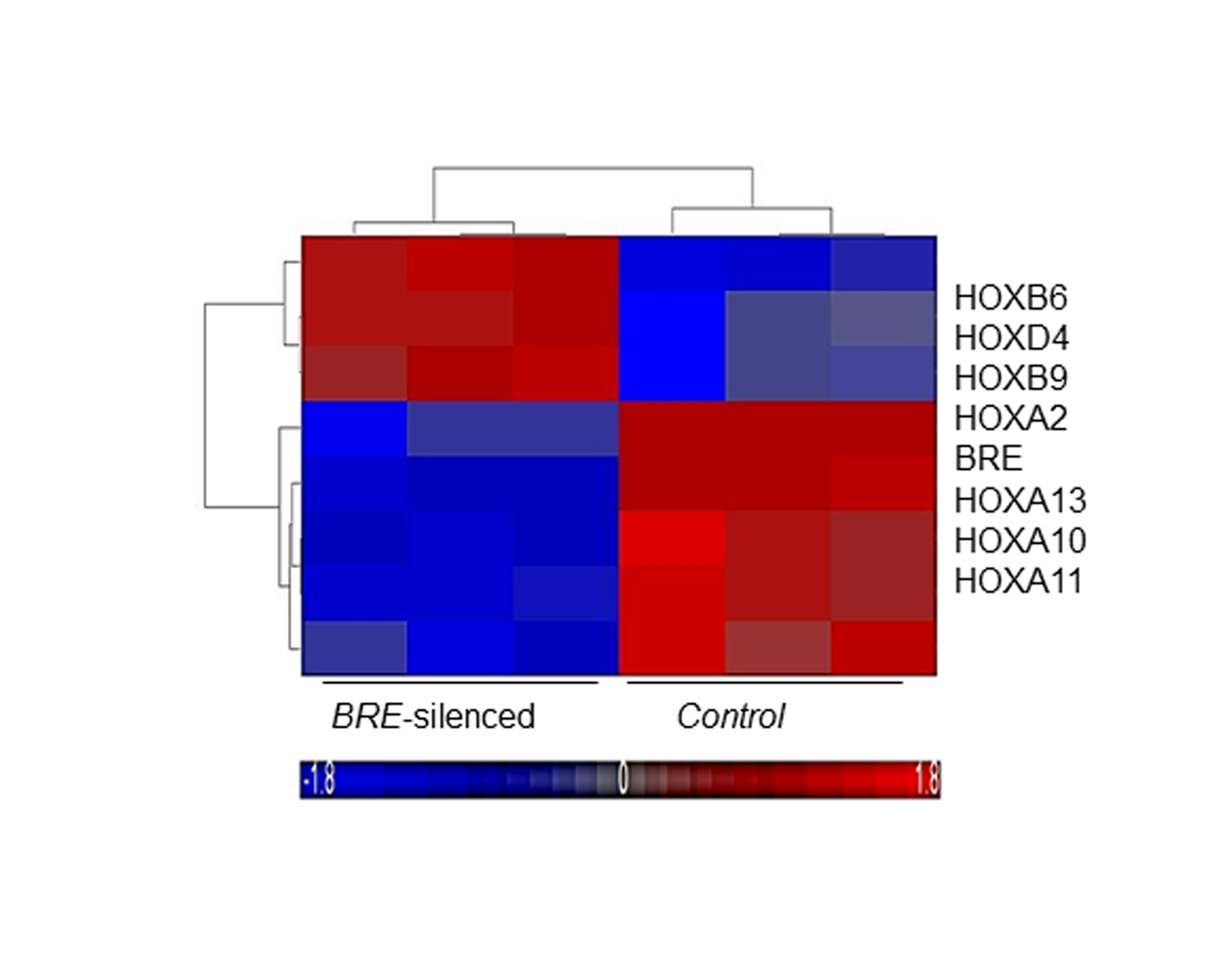

Supplement: Figure S7 — Microarray analyses of differentially expressed Hox genes in response to BRE -silencing. (A) Heat map displaying the patterns of differentially expressed Hox genes. The red boxes indicate that the gene signal is 36 higher than the background signal (grey boxes); whereas blue boxes indicate the signal is lower than background. (TIF) [file pone.0067896.s007.tif]

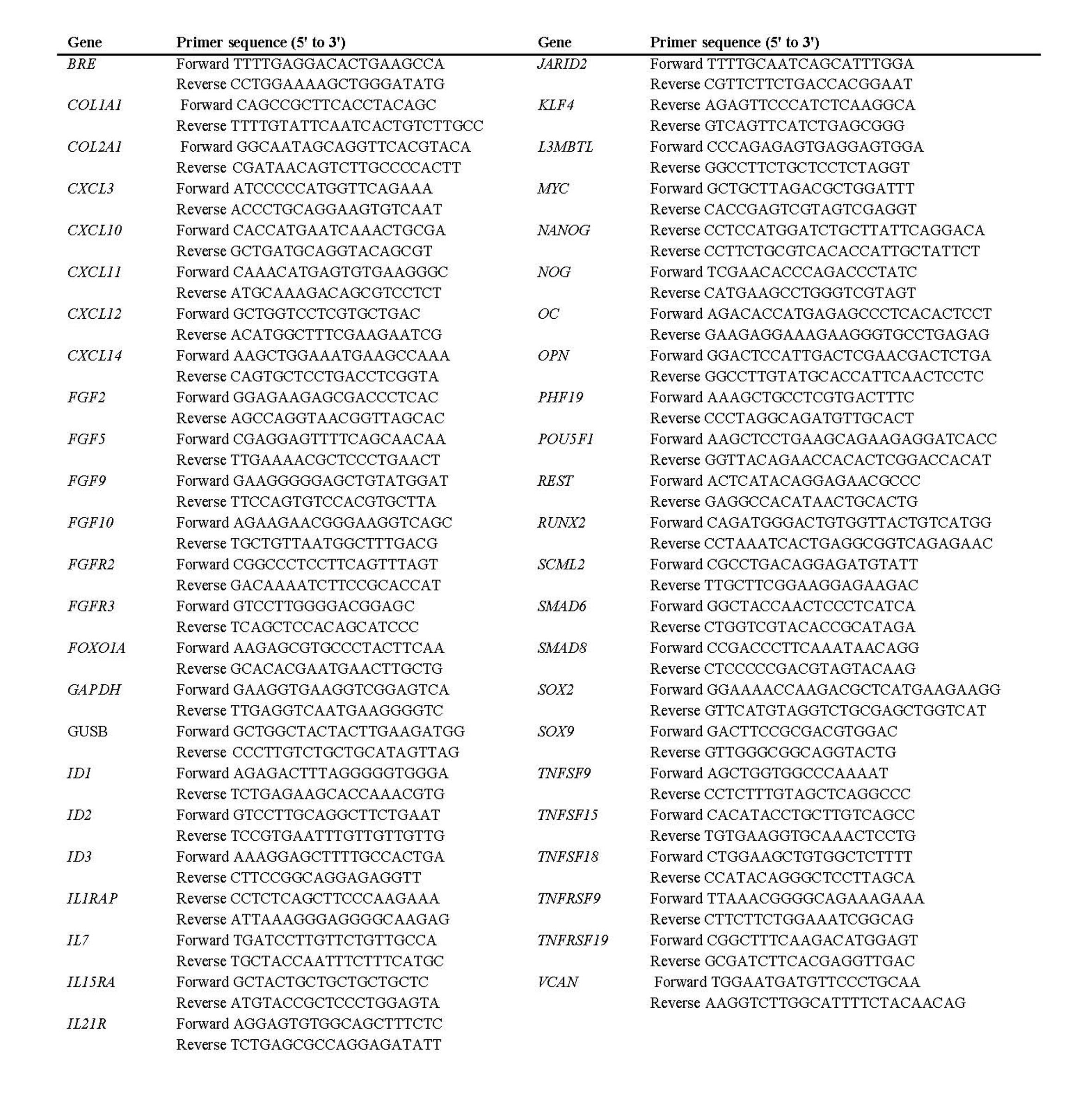

Supplement: Table S1 — The sequences of primers used in the RT-qPCR. (JPG) [file pone.0067896.s008.jpg]

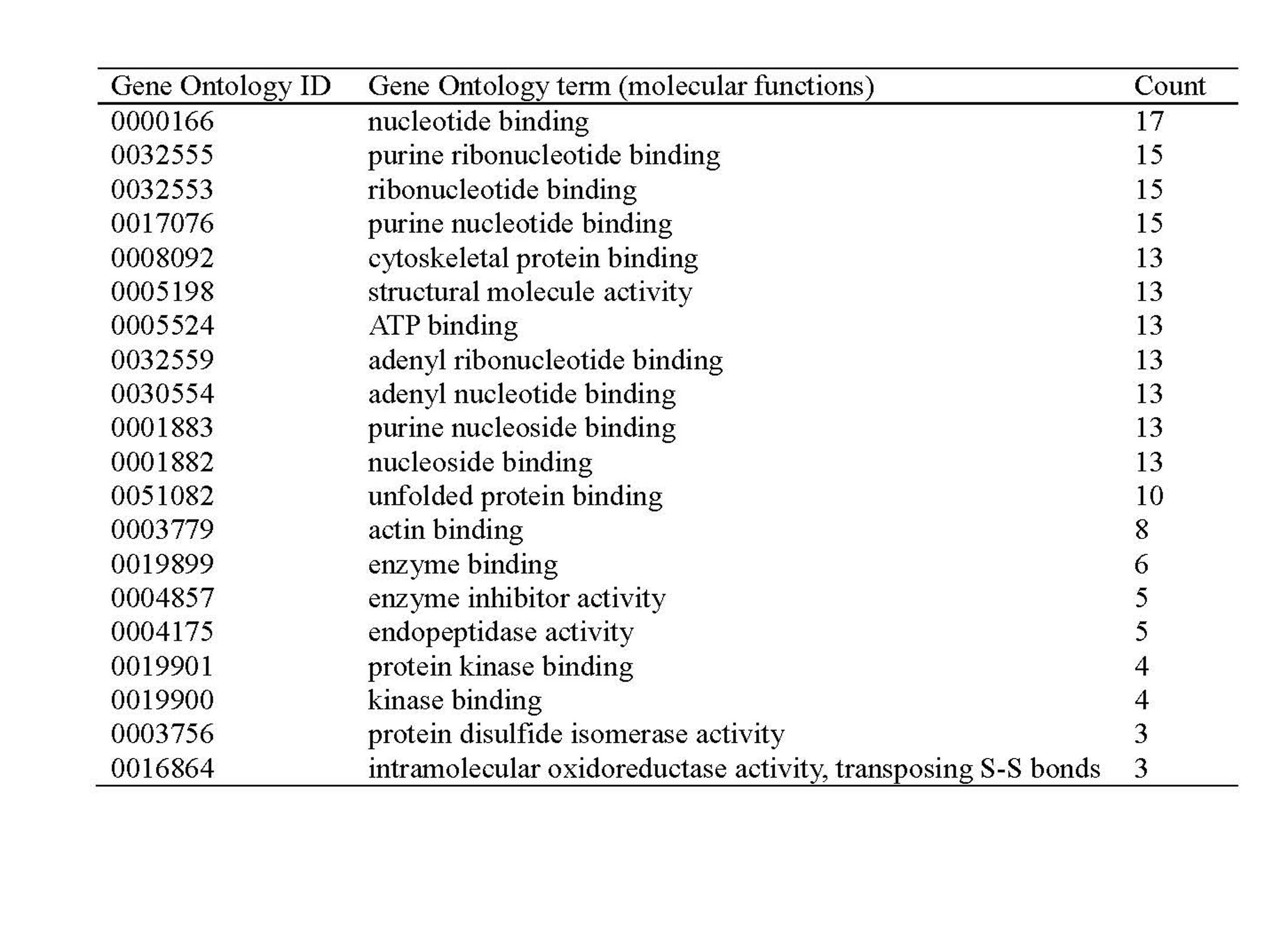

Supplement: Table S2 — The top 20 proteins identified by comparing BRE -silenced and control human umbilical cord perivascular cells, grouped based on molecular function using Gene Ontology tools. (JPG) [file pone.0067896.s009.jpg]

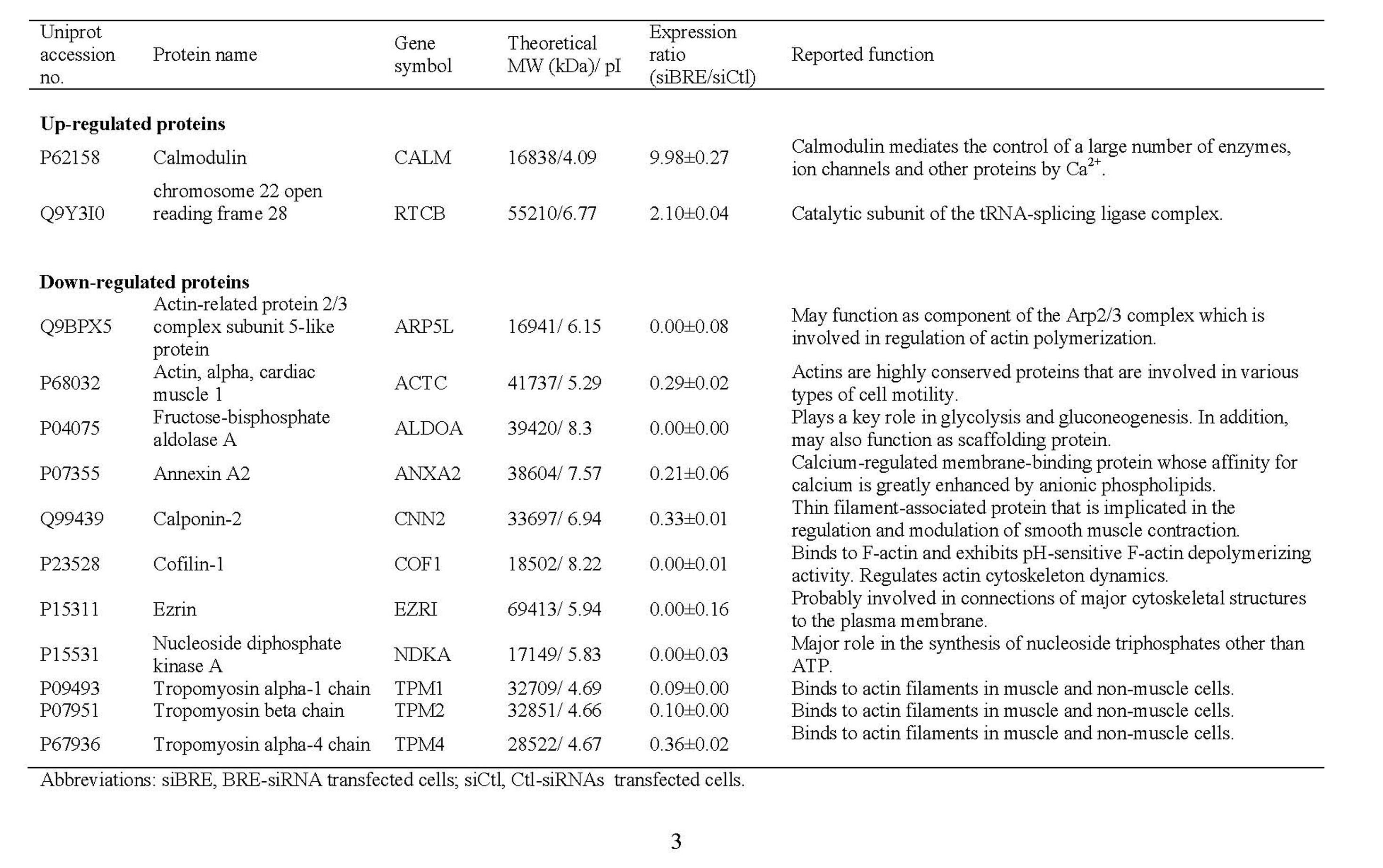

Supplement: Table S3 — Cytoskeletal binding proteins differentially expressed in BRE -silenced human umbilical cord perivascular cells. (JPG) [file pone.0067896.s010.jpg]

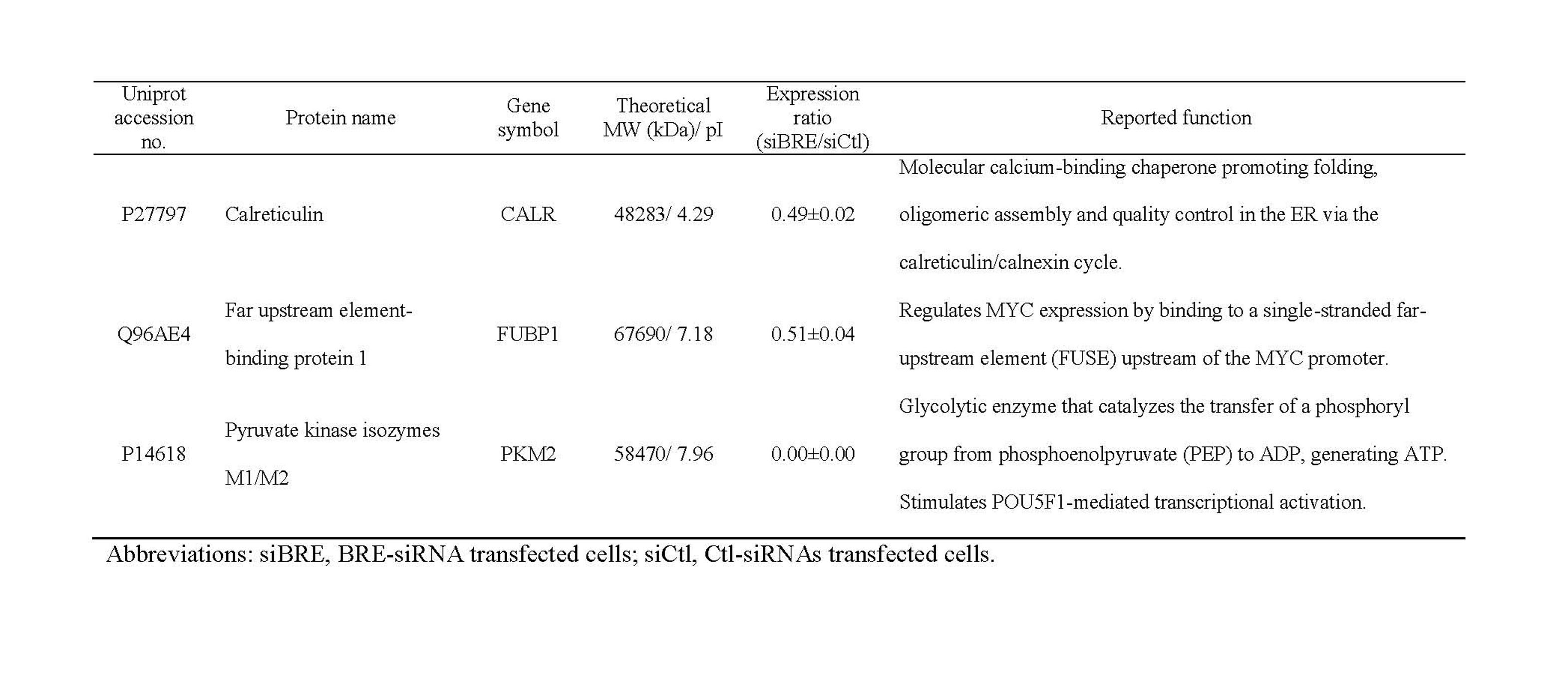

Supplement: Table S4 — Stemness-associated proteins differentially expressed in BRE -silenced human umbilical cord perivascular cells. (JPG) [file pone.0067896.s011.jpg]
